# Massively parallel identification of mRNA localization elements in primary cortical neurons

**DOI:** 10.1038/s41593-022-01243-x

**Published:** 2023-01-16

**Authors:** Samantha Mendonsa, Nicolai von Kügelgen, Sayaka Dantsuji, Maya Ron, Laura Breimann, Artem Baranovskii, Inga Lödige, Marieluise Kirchner, Meret Fischer, Nadja Zerna, Lucija Bujanic, Philipp Mertins, Igor Ulitsky, Marina Chekulaeva

**Affiliations:** 1 Max-Delbrück-Center for Molecular Medicine in the Helmholtz Association (MDC), Berlin Institute for Medical Systems Biology (BIMSB), Berlin, Germany; 2 Free University Berlin, Berlin, Germany; 3 Department of Immunology and Regenerative Biology and Department of Molecular Neuroscience, Weizmann Institute of Science, Rehovot, Israel; 4 Department of Genetics, Harvard Medical School, Boston, MA USA; 5 Core Unit Proteomics, Berlin Institute of Health at Charite-Universitätsmedizin Berlin and Max-Delbrück-Center for Molecular Medicine in the Helmholtz Association (MDC), Berlin, Germany

**Keywords:** Molecular neuroscience, RNA transport, Systems biology

## Abstract

Cells adopt highly polarized shapes and form distinct subcellular compartments in many cases due to the localization of many mRNAs to specific areas, where they are translated into proteins with local functions. This mRNA localization is mediated by specific *cis*-regulatory elements in mRNAs, commonly called ‘zipcodes’. Although there are hundreds of localized mRNAs, only a few zipcodes have been characterized. Here we describe a novel neuronal zipcode identification protocol (N-zip) that can identify zipcodes across hundreds of 3′ untranslated regions. This approach combines a method of separating the principal subcellular compartments of neurons—cell bodies and neurites—with a massively parallel reporter assay. N-zip identifies the let-7 binding site and (AU)_n_ motif as de novo zipcodes in mouse primary cortical neurons. Our analysis also provides, to our knowledge, the first demonstration of an miRNA affecting mRNA localization and suggests a strategy for detecting many more zipcodes.

## Abstract (web summary)

The authors established a method to map RNA localization sequences (zipcodes) and identified the let-7 site and AU repeat as new zipcodes in primary cortical neurons.

## Main

Delivery of mRNAs to specific subcellular locations is a key mechanism to produce localized pools of proteins. This process occurs in organisms as diverse as yeast, plants, insects and vertebrates (reviewed in ref. ^1^). It is particularly prominent in highly polarized cells, such as oocytes, migrating cells and neurons. For example, the development of the embryonic body axes in the *Drosophila* oocyte relies on the asymmetric localization of four maternal mRNAs: *gurken*, *bicoid*, *oskar* and *nanos* (reviewed in ref. ^1^). Neuronal functions also depend on specific patterns of mRNA localization to cell bodies (soma) and extensions (neurites). For example, in developing neurons, localization of β-actin mRNA to growth cones plays an essential role in axon guidance^2,3^.

The localization is thought to be mediated by *cis*-regulatory elements (‘zipcodes’) that are usually found in mRNA 3′ untranslated regions (UTRs)^4^. Zipcodes are bound by specific RNA-binding proteins (RBPs) that link their targets to transport machinery or regulators of mRNA stability and direct mRNAs to the sites of function. A few zipcodes and their bound RBPs have been described so far. Localization of β-actin is mediated by a 54-nucleotide (nt) zipcode, which targets mRNA to the cell periphery^5^. Zipcode-binding protein 1 (ZBP1) was identified as a binder of the *β-actin* zipcode, playing a role in both localization and translational control^6,7^. The cytoplasmic polyadenylation element (CPE) and its binding protein CPEB also facilitate transport to dendrites of several mRNAs, including *Map2* (microtubule-associated protein 2)^8^ and *Bdnf* (brain-derived neurotrophic factor)^9^. Localization of other transcripts with essential functions in neurites, such as *CaMKIIa* (Ca^2+^/calmodulin-dependent protein kinase II subunit α)^10^, *Arc* (activity-regulated cytoskeleton-associated protein)^11^ and *Mapt* (microtubule-associated protein tau)^8^, was also reported to depend on sequences in their 3′ UTRs.

High-throughput analyses have demonstrated specific localization patterns for hundreds to thousands of mRNAs in diverse organisms and cell types^12–25^. Presumably, many of these events rely on a similar mechanism, but, to date, only a few zipcodes have been characterized. Here we report the development of a method to systematically map neuronal zipcodes transcriptome-wide. Our approach combines a massively parallel reporter assay (MRPA) with the isolation of neuronal subcellular compartments: soma and neurites. We identify the let-7 binding site and (AU)_n_ motif as de novo zipcodes in mouse primary cortical neurons (PCNs). To our knowledge, our work provides the first demonstration of an miRNA affecting mRNA localization.

## Results

### Development of the neuronal zipcode identification protocol

**Fig. 1:**
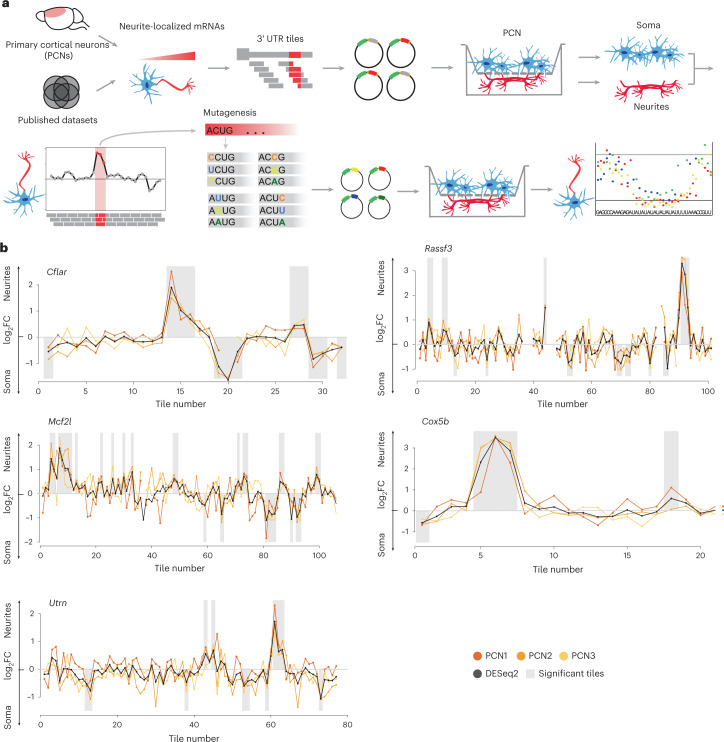
N-zip identifies neuronal zipcodes in PCNs. **a**, Scheme of N-zip. The method involves the following steps. (1) Integrative analysis is used to identify a group of transcripts localized to neurites in at least two neuronal localization datasets; fragments tiled across 3′ UTRs of these transcripts (tiles) are generated. (2) Tiles are cloned in 3′ UTRs of lentiviral vector, between adapter sequences, to generate a pooled reporter library; to restrict the expression of the library to neurons, a neuron-specific synapsin promoter is used. (3) The resulting library is delivered into PCNs grown on a microporous filter, separating soma from neurites. (4) Soma and neurites are isolated, and RNA-seq libraries are prepared from RNA fragments flanked by adapters. (5) Tiles that are sufficient for the localization of RNAs to neurites are identified. (6) An extensive mutagenesis of these tiles is performed, and steps 2–5 are repeated to map the specific sequences that serve as zipcodes. **b**, N-zip identifies 3′ UTR fragments driving RNA localization to neurites of PCNs. Specific examples of identified tiled fragments that mediate localization to neurites are shown. Enrichment of a given tile (log_2_-transformed fold change neurites/soma (log_2_FC) ≥ 1, adjusted *P* < 0.1) (*y*) is plotted against tiled fragment number (*x*). Neurite/soma ratios for individual biological replicates (shades of yellow: PCN1, PCN2 and PCN3) and ratios computed by DESeq2 based on all replicates (black line) are shown. Shaded regions indicate tiles with significant enrichment (*P* < 0.05) in one of the subcellular compartments. The gene name is shown above each plot.

**Extended Data Fig. 1:**
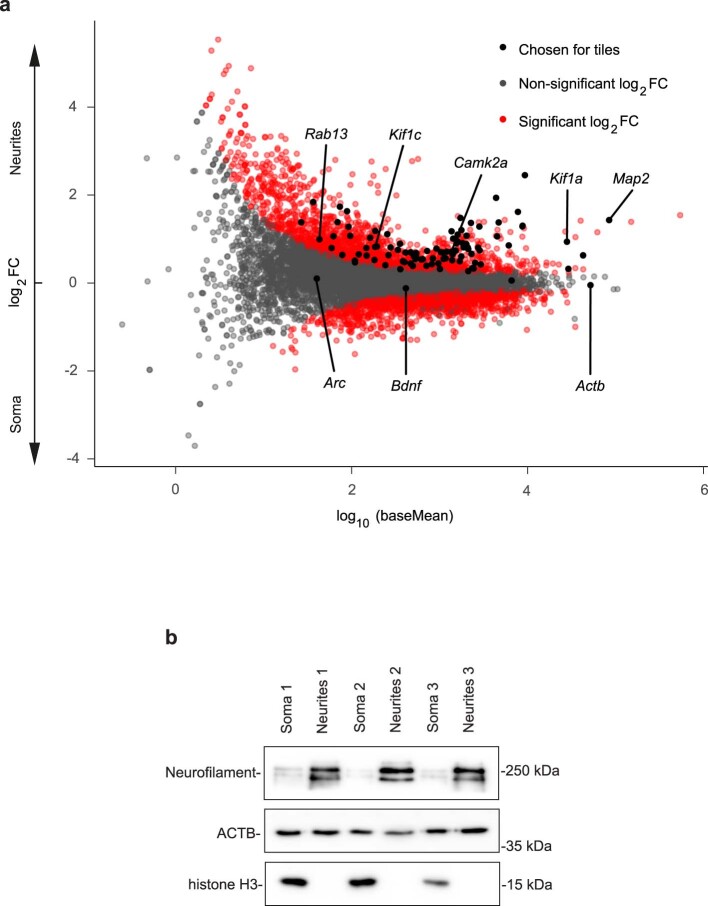
Analysis of local transcriptome of mouse primary cortical neurons. (**a**) Mean abundance (MA) plot showing enrichment of transcripts in neurites versus soma (Y), plotted against their mean abundance (X). Transcripts are colored by significance (red: adjusted p-value < 0.05) and selection for the N-zip library (black). Selected neuronal mRNAs are labeled. (**b**) Neurites and soma of primary cortical neurons are efficiently separated with the microporous membrane. Neurons were grown on a microporous membrane so that cell bodies stay on the top and neurites grow through the pores on the lower side, as described earlier^25^ (see Methods for details). Protein lysates prepared from isolated neurite and soma fractions were analyzed by western blotting with antibodies against histone H3 (soma marker) and Neurofilament (NF, neurite marker). Three independent biological replicates are shown. ACTB was used as a normalization control. Source data

To perform an unbiased transcriptome-wide analysis of zipcodes, we developed the neuronal zipcode identification protocol (N-zip). This combines MPRA^26^ with a neurite/soma fractionation scheme established previously^24,25,27^ (Fig. 1a). As the input for N-zip, we selected 99 transcripts localized to neurites in mouse PCNs (Extended Data Fig. 1a) and at least one other published dataset generated from primary neurons: dorsal root ganglia, cortical, hippocampal or motor neurons^20–22,28–31^. To narrow down regions containing potential zipcodes, we designed a pool of 4,813 oligos, 75–110 nt in length, tiled across 3′ UTRs of selected neurite-enriched transcripts with 15–25-nt offset (Supplementary Table 1). The oligos were cloned into the 3′ UTR of GFP reporter, and the resulting pooled lentiviral library was delivered into PCNs. Infected neurons were cultured on a microporous membrane so that soma stayed on top of the membrane, and neurites grew through the pores on the lower side^25^. We isolated neurites and soma, carried out RT–PCR and prepared amplicon sequencing libraries, referred to as N-zip libraries. Western blotting confirmed the separation efficiency between neurites and soma (Extended Data Fig. 1b).

The term ‘mRNA localization’ has been used in two different ways: to signify the mere presence of mRNA in neurites and as an enrichment of mRNA in neurites versus soma. Because enrichment points to active localization, we focus on transcripts enriched in neurites. Our analysis of triplicate N-zip libraries identified 65 neurite-localized tiled fragments or tiles (Supplementary Table 1 and Fig. 1b). These tiles mapped to 33 out of 99 transcripts included in the library. For example, we detected a neurite localization of tiles 7–10 from *Mcf2l* (*Mcf2l*-7–10), which encodes the guanine nucleotide exchange factor for CDC42 and RHOA. This protein mediates the formation and stabilization of the glutamatergic synapses and is associated with intellectual disability and autism^32^. Similarly, we observed a neuritic enrichment of *Utrn-*61, which encodes a component of a dystrophin glycoprotein complex. This complex links the actin cytoskeleton to the extracellular matrix and plays a role in forming neuromuscular junctions^33^. Among other transcripts with a neurite-localized tile were cell growth and survival regulators *Rassf3* (ref. ^34^) and *Cflar*^35^ as well as *Cox5b*, which encodes a mitochondrial enzyme.

**Extended Data Fig. 2:**
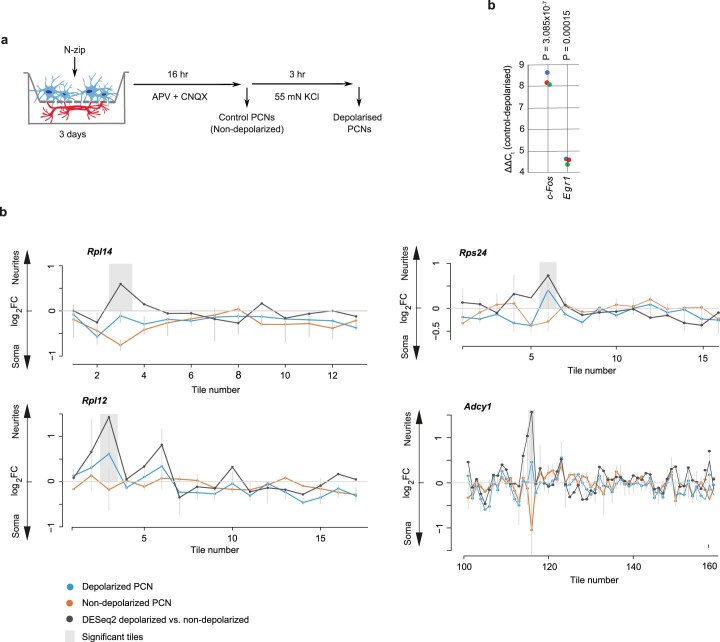
Depolarization of primary cortical neurons regulates localization of selected 3’UTR fragments to neurites. (**a**) Scheme showing depolarization of primary cortical neurons using KCl. (**b**) RT-qPCR shows an efficient upregulation of depolarisation markers *c-Fos* and *Egr1* in primary cortical neurons upon KCl treatment. Differences in *c-Fos* and *Egr1* expression levels between depolarized and control (non-depolarized) neurons (ΔΔC_t_) are plotted on (*Y*) as individual biological triplicates (colors dots, n = 3). *Gapdh* was used for normalization. The statistical significance of differences between KCl-treated and control samples was computed by two-sided t-test, and p-values are shown on the plot. (**c**) N-zip identifies 3’UTR fragments driving mRNA localization to neurites of primary cortical neurons upon depolarization. Specific examples of identified tiled fragments that mediate localization to neurites are shown. The data are presented as in Fig. 1b. The gene names are shown above the plot. Localization in depolarized neurons (blue line) and control non-depolarized neurons (orange line) is shown. The differential localization (log_2_FC neurites vs. soma) between depolarized and non-depolarized neurons was analyzed with DESeq2 (black line). Shaded in grey are tiles that show a statistically significant shift in localization to neurites upon depolarization. Source data

Neural stimulation was reported to enhance localization of certain mRNAs (reviewed in ref. ^1^). Such stimulation modulates the depolarization of neurons; therefore, we decided to analyze how depolarization affects localization of the N-zip reporter library. For that, we treated PCNs, grown on a microporous filter, with potassium chloride (KCl) (Extended Data Fig. 2a). As a proof that cells responded to the depolarizing stimulus, we showed an increase in levels of *c-Fos* and *Egr1*, whose expression is triggered by depolarization^36^ (Extended Data Fig. 2b). We then prepared N-zip libraries from such depolarized and control non-depolarized neurons. Depolarization affected localization of 123 tiles from 51 transcripts (DESeq2 adjusted *P* < 0.05 for difference in neurites/soma ratios), out of which 55 became more neurite-enriched and 68 became more soma-enriched. Curiously, among them were transcripts encoding ribosomal proteins and *Adcy1* (adenylyl cyclase type 1), which plays an essential role in synaptic plasticity^37^ (Extended Data Fig. 2c).

**Extended Data Fig. 3:**
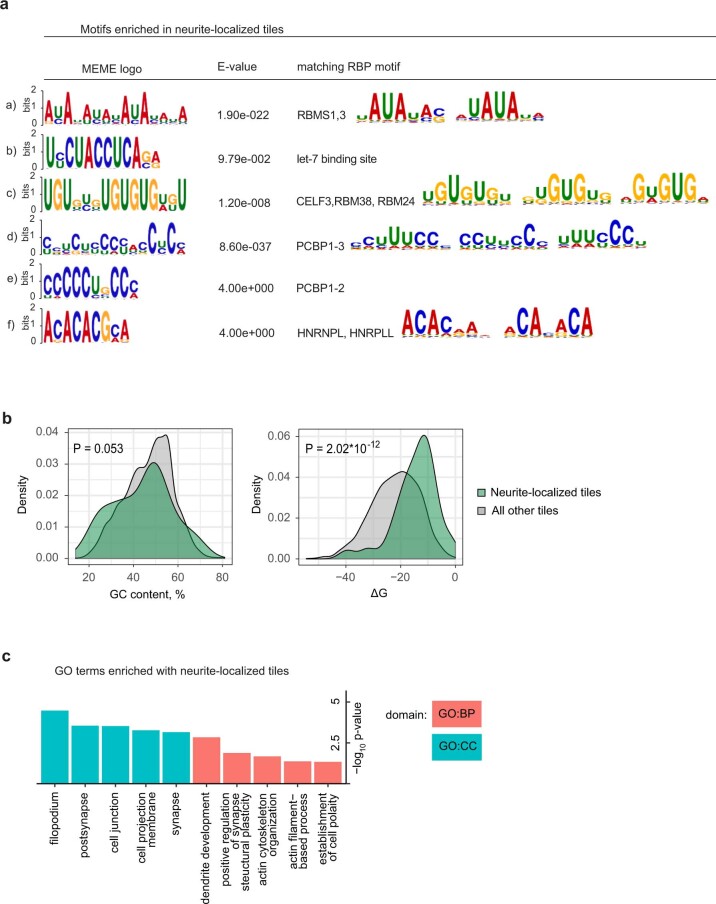
3’UTR fragments localized to neurites of primary cortical neurons are enriched in specific motifs and map to the genes associated with synaptic functions and structures. (**a**) Motifs enriched in neurite-localized tiles in primary cortical neurons. Motif discovery was done with XTSREME tool of the MEME suite on all neurite-localized tiles in N-zip (log_2_FC neurites/soma ≥ 1, adjusted p-value < 0.1), using all fragments included in N-zip library (Supplementary Table 1) as a background. As a measure of the statistical significance, E-values computed by XTSREME^38^ are provided. RBPs with matching motifs are shown. P-values were computed with DEseq2, which uses Wald test; Benjamini-Hochberg procedure was used for correction of p-values for multiple testing. (**b**) Neurite-localized tiles in N-zip have an average CG content and a low tendency to form secondary structures. Histograms showing the CG content (left) and the Gibbs free energy change (ΔG) as a measure of secondary structure formation (right) for neurite-localized tiles (green) and all tiles included in N-zip (grey). For ΔG calculation, we used RNAfold^100^. P-values were computed using two-sided Wilcoxon rank-sum test. (**c**) Transcripts with neurite-localized tiles are linked with synaptic functions, cytoskeleton and cell polarity. Gene Ontology (GO) terms overrepresentation analysis was performed on transcripts with neurite-localized tiles (log_2_FC neurites/soma ≥ 1, adjusted p-value < 0.1, Supplementary Table 1), using gProfiler2 (ref. ^98^). P-values were computed as in (**a**). All transcripts included in N-zip library were used as a reference. The top 5 GO terms with the lowest p-values are plotted.

We performed a comprehensive motif analysis on neurite-localized tiles using the XSTREME tool from the MEME suite^38^. Among identified motifs, there were UYCUACCUCAGA (Y: pyrimidine, C or U), AU-rich, GU-rich and C-rich motifs (Extended Data Fig. 3a). Further sequence analysis showed that neurite-localized tiles have no bias in GC content and a low tendency to form secondary structures (Extended Data Fig. 3b). Curiously, Gene Ontology (GO) term analysis showed that transcripts with neurite-localized tiles are linked with local neuronal structures and processes, such as synapse, actin cytoskeleton and cell polarity (Extended Data Fig. 3c).

**Fig. 2:**
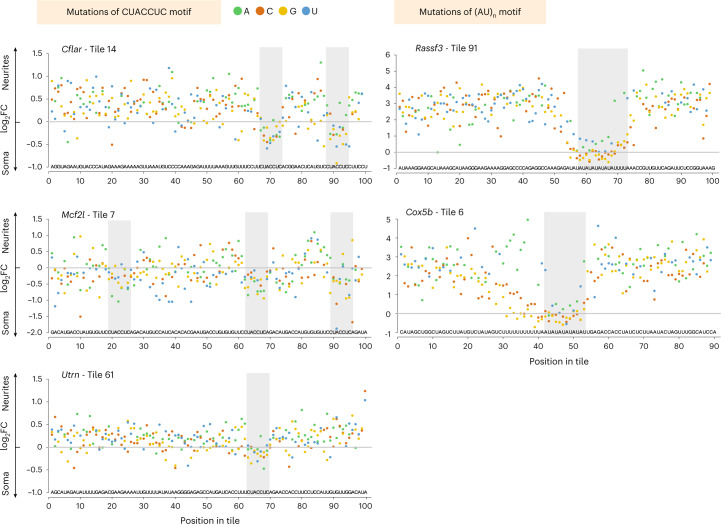
N-zip combined with mutagenesis maps motifs driving mRNA localization to neurites of PCNs. Selected localized tiled fragments were mutagenized and used for the secondary N-zip in PCNs as shown in Fig. 1a (step ‘Mutagenesis’). Specific examples of mutated motifs that mediate localization to neurites are shown. The data are presented as in Fig. 1b. The initial sequence of mutagenized fragment is shown above the *x* axis, and introduced point mutations are indicated with green (A), orange (C), yellow (G) and blue (U) dots. The gene name and tile number are shown above the plot.

To fine-map the sequences that mediate localization, we performed extensive mutagenesis of 16 neurite-localized fragments. We generated a secondary N-zip library with 6,266 sequences (Fig. 1a). In these cases, we (1) introduced every possible single point mutation; and (2) mutated G ↔ C and A ↔ U within 2-nt, 5-nt and 10-nt windows (Supplementary Table 2). This analysis identified two specific motifs required for localization to neurites: CUACCUC and (AU)_n_ (Fig. 2). Notably, both motifs were also identified in our motif analysis of the tiled library (Extended Data Fig. 3a).

**Extended Data Fig. 4:**
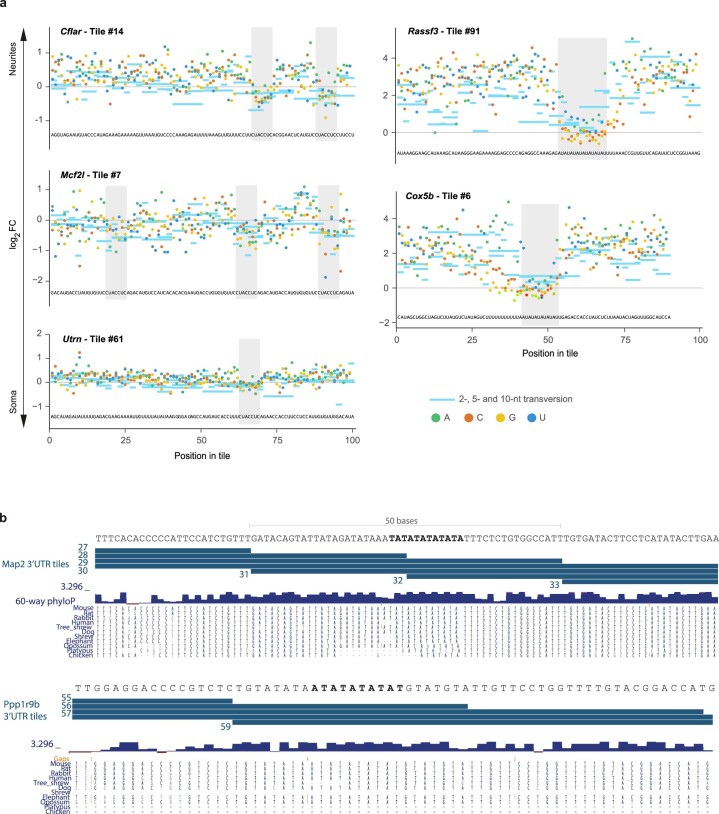
Elements mediating RNA localization in primary cortical neurons. (**a**) N-zip combined with mutagenesis of 2-nt, 5-nt and 10-nt windows maps motifs driving mRNA localization to neurites of primary cortical neurons. Each horizontal line indicates the window that was mutagenized and the corresponding neurites/soma ratio of the mutated sequence. Within each window, we mutated G ↔ C and A ↔ U. The data are presented as in Fig. 2. The gene name and tile number are shown above the plot. (**b**) Conservation of selected (AU)_n_ stretches across species. UCSC genome browser view of *Map2* and *Ppp1r9b* 3’UTRs with (AU)_n_ stretches. The mouse sequence for each stretch and the positions of N-zip tiles are shown on the top. The lower track shows the PhyloP conservation scores and the sequences of the corresponding stretches from other mammals.

Mutations in CUACCUC shifted the localization of *Cflar*-14, *Mcf2l*-7 and *Utrn*-61 toward soma. Any mutation of (AU)_8_ in *Rassf3-91* markedly reduced neurite enrichment. Similarly, the *Cox5b-6* (AU)_6_ motif was essential for localization, with contributions from flanking U and A bases and from an additional (U)_11_ stretch. This latter region could tolerate mutations to A but not to G or C. The results of single point mutations were confirmed by mutagenesis of 2-nt, 5-nt and 10-nt windows (Extended Data Fig. 4a).

**Extended Data Fig. 5:**
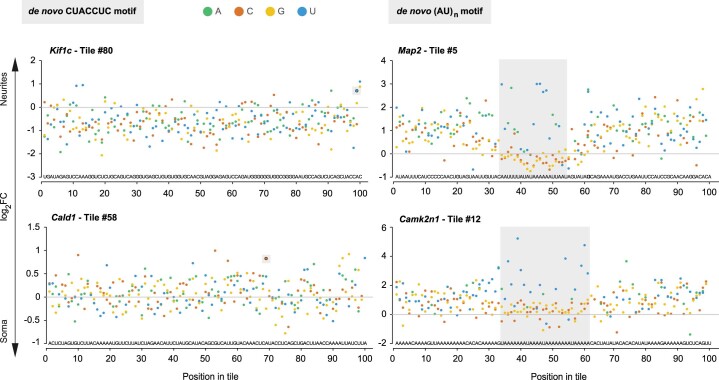
Introduction of *de novo* identified localization motifs into heterologous context drives their localization to neurites. Specific examples of N-zip fragments where either let-7 binding site or (AU)_n_ were introduced by chance during the mutagenesis step. The data are presented as in Fig. 2. The gene name and the number of the mutagenized tile are indicated above the plot. Neurite/soma ratios for indicated fragments with single point mutations are shown (Y), with the initial sequence of the mutagenized fragment displayed on (X) and the point mutations indicated with green (A), orange (C), yellow (G) and blue (U) dots. Shaded regions indicate fragments with significant enrichment (P < 0.05) in one of the subcellular compartments.

In both the original and the secondary library, (AU)_n_ was associated with neurite localization for *n* ≥ 6. In the original library, these motifs were also found in *Map2*-29–32, *Ppp1r9b*-56–59, *Shank3*-58–60 and *Tmcc2*-36–39 tiles, and some showed conservation in other mammals (Extended Data Fig. 4b). Curiously, in some cases, our mutagenesis introduced CUACCUC and (AU)_n_ stretches into heterologous sequences (Extended Data Fig. 5). These artificially created motifs resulted in neuritic localization of the fragment, showing that these motifs are not only necessary but also sufficient for localization to neurites.

### Let-7 directs localization of its target mRNAs to neurites

**Fig. 3:**
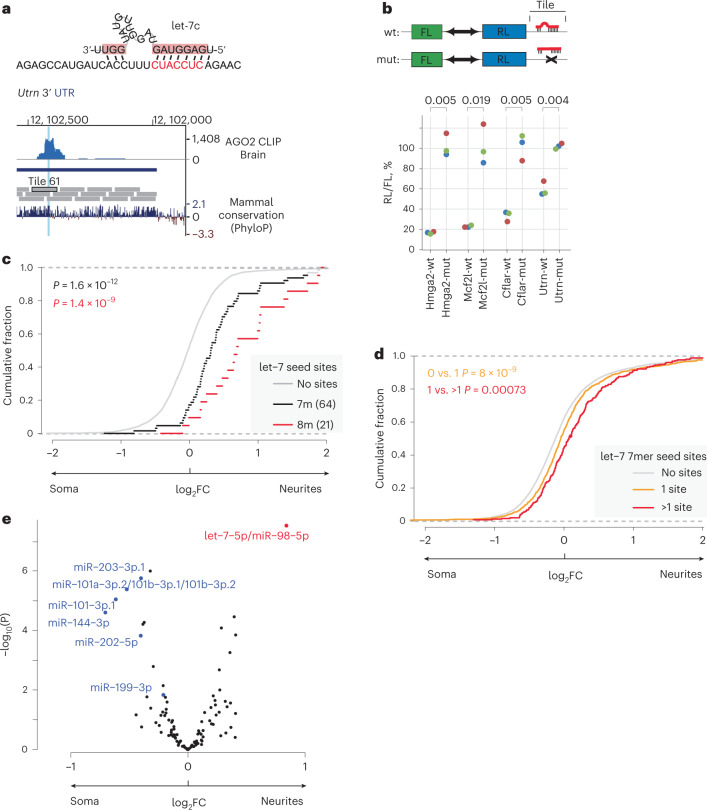
Let-7 binding sites direct mRNA localization to neurites in PCNs. **a**, Neurite-localized motif CUACCUC is a binding site for let-7. Top: base-paring between let-7c and *Utrn-*61 tile predicted by mfold^80^. Bottom: UCSC genome browser view showing coverage of AGO2 HITS-CLIP reads in mouse brain^41^ (upper track) around let-7 seed in *Utrn-*61 (highlighted in blue). Lower track shows sequence conservation (PholyP). **b**, Validation of functionality of let-7 sites in *Cflar-*14, *Mcf2l-*7 and *Utrn-*61 in luciferase reporter assay. Indicated reporters were transfected in HeLa-rtTA cells: *Renilla* luciferase (RL) and firefly luciferase (FL) are produced from the same vector, with indicated N-zip tiles, either with wild-type (wt) or with mutated (mut) let-7 sites, inserted downstream of RL. As a positive control, analogous reporters with *Hmga2* 3′ UTR (hmga2-wt and hmga2-mut) were used. For each reporter pair, RL activity was normalized to that of FL and presented as a percentage of luciferase activity produced by mutated reporter. Individual biological triplicates (colored dots, *n* = 3) are plotted. Statistical significance of differences between wild-type and mutated reporters were computed by two-sided *t*-test and shown on the plot. **c**, N-zip mRNAs with let-7 sites are enriched in neurites of PCNs. Cumulative distribution function (CDF) showing fractions of mRNAs (*y*) plotted against neurite/soma enrichment (*x*) for transcripts with no let-7 sites (gray), at least one 7mer (black) or 8mer let-7 site (red). The number of detected tiles with indicated let-7 sites is shown in the legend. *P* values were computed with two-sided Wilcoxon rank-sum test. **d**, Endogenous mRNAs with let-7 sites are enriched in neurites of PCNs. The data are plotted, and *P* values were computed as in **c** for transcripts with no (gray), one (yellow) or more than one (red) let-7 sites. **e**, Let-7 sites are enriched in neurite-localized mRNAs in PCNs. Volcano plot showing the mean neurite/soma enrichment of tiles containing 7mer matches to the seeds of individual miRNA families in N-zip mRNAs. miRNAs with statistically significant enrichment in one of the compartments (*P* < 0.05, two-sided *t*-test) are labeled in red (neurite-enriched) and blue (soma-enriched). Source data

Analysis of miRbase^39^ showed that the CUACCUC motif, which we identified as a de novo zipcode in N-zip (Fig. 2), represents the binding site for the seed of the let-7 miRNA family (Fig. 3a, top). miRNAs regulate gene expression by pairing with complementary sites in their target mRNAs; this recruits a complex of proteins that destabilizes the mRNAs^40^. The miRNA seed is a conserved sequence at positions 2–7 from the miRNA 5′ end, which binds to target mRNAs via a perfect base-pairing. Indeed, every point mutation in CUACCUC affected localization of tiles containing this motif (Fig. 2b). This is to be expected from an miRNA seed site but not from a consensus RBP motif. Furthermore, in mouse brain, the position of CUACCUC in *Utrn-61* matched the summit of a cross-linking immunoprecipitation (CLIP) peak for AGO2 (ref. ^41^), which is the core component of the miRNA repression complex (Fig. 3a, bottom).

To confirm that the let-7 binding sites in *Cflar*-14, *Mcf2l*-7 and *Utrn*-61 are functional, we performed a luciferase reporter assay (Fig. 3b). As a positive control, we used a reporter bearing the *Hmga2* 3′ UTR (Hmga2-wt), a validated let-7 target^42^. Endogenously produced in HeLa cells, let-7 repressed Hmga2-wt >5-fold compared to a mutant version lacking let-7 sites (Hmga2-mut). We generated analogous luciferase reporters bearing the tested tiles in their 3′ UTRs (Mcf2l-wt, Cflar-wt and Urtn-wt). As negative controls, we mutated let-7 seeds in the tested regions (Mcf2l-mut, Cflar-mut and Utrn-mut). Compared to the mutated versions, the Mcf2l-wt with three let-7 sites was repressed about five-fold, the Cflar-wt with two let-7 sites about four-fold and the Utrn-wt with a single let-7 site about 1.7-fold. These data confirmed that the let-7 sites in the analyzed tiles are functional.

We next wondered whether other let-7 targets localize to neurites. To test this, we examined the frequency of let-7 sites across differentially localized transcripts in our N-zip libraries. Consistent with the role of let-7 binding sites in mRNA localization, we observed an enrichment of transcripts bearing let-7 7mer seeds in neurites (black line, Fig. 3c) compared to transcripts without let-7 seeds (gray line). The effect was even stronger for extended 8mer seeds (red line).

Next, we examined if let-7 sites also contribute to the localization of endogenous mRNAs. We detected an enrichment of let-7 site-bearing endogenous transcripts in neurites, the degree of which depended on the number of let-7 sites (gray line: no sites, yellow line: one site, red line >1 site; Fig. 3d and Supplementary Table 3). This let-7 site-dependent shift in localization was less profound for endogenous mRNAs than for N-zip reporters, probably because other regions in endogenous full-length 3′ UTRs contribute to their localization.

**Fig. 4:**
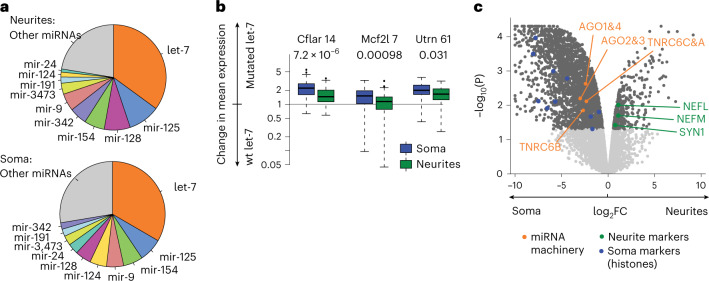
Let-7 functions by preferentially destabilizing its target mRNAs in soma of PCNs. **a**, Let-7 is the most abundant miRNA in PCNs. Pie charts showing the percentage of different miRNAs in neurites and soma of PCNs, determined by small RNA-seq. **b**, Mutations of let-7 binding sites lead to a stronger increase of N-zip mRNA reporter levels in soma. Box plots showing changes in the normalized expression levels between tiles that contain intact let-7 seed sites (wt let-7) and tiles in which let-7 seeds were mutated (mutated let-7). In the mutated tiles, at least one let-7 seed site was lost. The data are shown separately for each tile (as indicated on *x*) and each subcellular compartment (green: neurite, blue: soma). *P* values, computed with two-sided Wilcoxon rank-sum test, are shown above the plots (*n* = 3 independent biological replicates). Box plot elements, here and further, unless otherwise specified: center line, medium; box limits, upper and lower quartiles; whiskers, 1.5× interquartile range; points, outliers. **c**, Proteomic analysis of isolated soma and neurites indicates somatic enrichment of the core components of the miRNA repression complex (AGO1, 2, 3 and 4; TNRC6A, B and C, orange). Volcano plot showing −log_10_ *P* values (*y*) plotted against log_2_FC of iBAQ values, normalized to GAPDH in each compartment. *P* values were computed with limma’s eBayes function (Methods) and adjusted for multiple testing using the Benjamini–Hochberg method. Significant values (FDR < 5%) are shown in dark gray and non-significant in light gray. Neurofilaments (NEFL and NEFM) and synapsin (SYN1) were used as neurite markers (green) and histones as soma markers (blue) to confirm the efficiency of the separation of soma and neurites.

**Extended Data Fig. 6:**
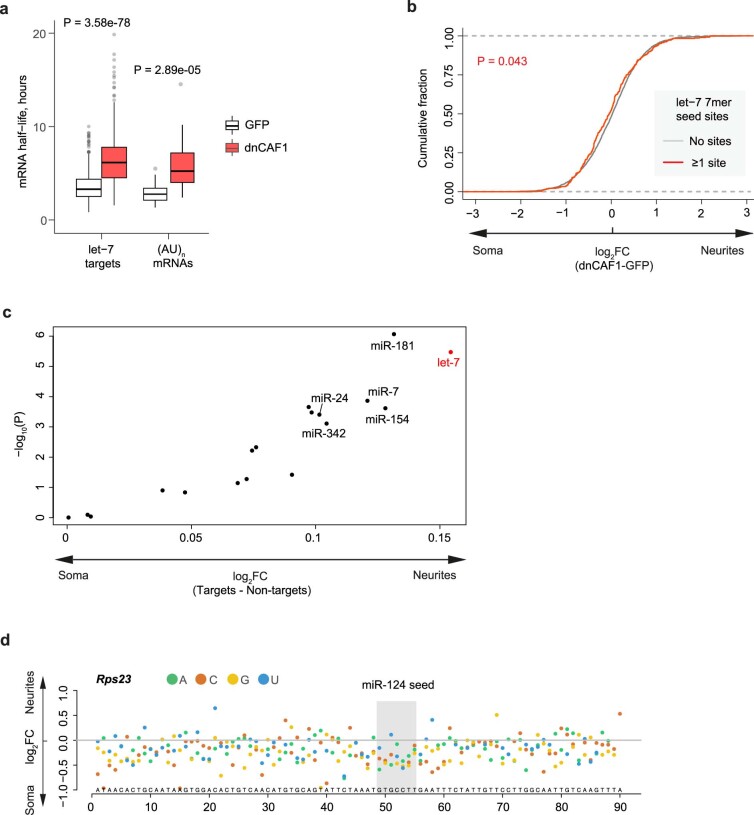
Effects of miRNAs on mRNA localization in primary cortical neurons. (**a**) Functional depletion of CAF1 via dnCAF1 expression leads to stabilization of let-7 targets and (AU)_n≥6_-containing mRNAs. Boxplots showing the distribution of half-lives (*Y*), measured by SLAM-seq, for endogenous mRNAs bearing at least one let-7 seed site or (AU)_n≥6_ stretch in their 3’UTR. The data are shown for primary cortical neurons expressing dnCAF1 (red boxes) or GFP (white boxes, negative control). P-values were computed with two-sided Wilcoxon rank-sum test; n = 3 independent biological replicates. (**b**) mRNA stabilization with dnCAF1 shift localization of let-7 targets towards soma. CDFs showing fractions of endogenous mRNAs with (red) and without let-7 sites (grey), as measured by mRNA-seq (*Y*), plotted against changes in neurite/soma enrichment upon expression of dnCAF1. Two-sided Kolmogorov-Smirnov test was used to estimate the significance. (**c**) The difference in neurites vs. soma enrichment (log_2_FC) between targets and non-targets of indicated miRNAs (X) is plotted against -log_10_ of P-values (Y). The data are provided for the top 20 miRNAs expressed in primary cortical neurons, as shown in Fig. 4a. miRNAs with statistically significant enrichment in neurites (P < 0.05, computed by two-sided t-test) are labeled in red. (**d**) Mutation of miR-124 site in *Rps23* tile affects its localization. Enrichment of *Rps23* tile (log_2_FC) between subcellular compartments (Y) is plotted for individual point mutants of the tile (X). The initial sequence of the mutagenized fragment is shown above X axis, and the introduced point mutations are indicated with green (A), orange (C), yellow (G) and blue (U) dots. Shaded regions indicate tiles with a significant change in enrichment (P < 0.05). P = 4.11×10^–6^ between the *Rps23* mutated tiles where the miR-124 seed was mutated and all other mutated *Rps23* tiles (two-sided Wilcoxon rank-sum test).

Our global analysis of miRNA seeds in N-zip libraries revealed that let-7 sites were enriched compared to sites of other miRNAs in neurites (Fig. 3e). To investigate the potential underpinnings of this specificity, we analyzed miRNA expression levels by small RNA sequencing (RNA-seq). We found that let-7 is the most abundant miRNA in PCNs (>30% of all miRNA reads; Fig. 4a and Supplementary Table 4), explaining the preferential influence of let-7 on the neurite-enriched transcriptome. Given the role of miRNAs in mRNA stability, we compared levels of N-zip reporters with let-7 sites with those in which let-7 sites had been mutated. Mutations of let-7 sites stabilized N-zip reporters, and this effect was stronger in soma than in neurites (Fig. 4b, compare blue and green boxes). These data suggest that let-7 promotes the enrichment of its targets in neurites by destabilizing them more potently in soma. To experimentally test this hypothesis, we perturbed mRNA degradation by expressing a dominant negative mutant of deadenylase CAF1 (dnCAF1)^43^. Expression of dnCAF1 led to stabilization of let-7 targets, compared with control GFP-expressing PCNs (Extended Data Fig. 6a, compare white and red boxes). We then isolated soma and neurites and analyzed changes in mRNA localization upon dnCAF1 expression by RNA-seq. Consistently with our model that let-7 promotes the enrichment of its targets in neurites through regulation of their stability, let-7 targets shifted to soma, compared with transcripts lacking let-7 sites (Extended Data Fig. 6b).

Because levels of let-7 were similar in neurites and soma, we next used proteomics to examine the levels of proteins involved in miRNA-mediated regulation. This analysis showed that the core components of the miRNA repression complex (AGO and TNRC6 family members) are enriched in soma (Fig. 4c, orange, and Supplementary Table 5). These data explain a higher let-7 activity in soma and the enrichment of let-7 targets in neurites.

**Fig. 5:**
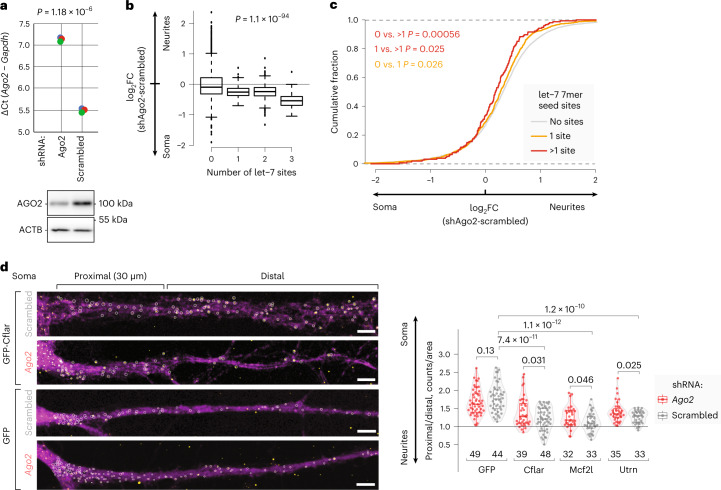
Localization of let-7 targets to neurites requires AGO2. **a**, Efficient AGO2 depletion in PCNs. *Ago2* expression levels in *Ago2*-depleted and control scrambled shRNA samples were quantified by RT–qPCR, normalized to *Gapdh*, and plotted on *y* as individual biological triplicates (colored dots, *n* = 3). *P* value was computed by two-sided *t*-test. Western blotting against AGO2 and ACTB (loading control) is shown below the plot. **b**, *Ago2* knockdown shifts N-zip reporters with let-7 sites toward soma. Box plots showing changes in neurite/soma enrichment between *Ago2*-depleted and scrambled samples (*y*) as a function of the number of let-7 sites in the N-zip reporter tiles (*x*). *P* values were computed using two-sided Wilcoxon rank-sum test (*n* = 3 independent biological replicates). **c**, *Ago2* knockdown in PCNs shifts endogenous let-7 targets toward soma. Cumulative distribution functions (CDFs) showing fractions of endogenous mRNAs with no let-7 sites (gray), one let-7 site (yellow) and more let-7 sites (red) (*y*), plotted against changes in neurite/soma enrichment upon *Ago2* knockdown (*x*). *P* values were computed using two-sided Wilcoxon rank-sum test. **d**, smiFISH validates the role of AGO2 in the localization of let-7 targets. PCNs were transduced with either *Ago2*-targeting (red) or scrambled shRNAs (gray) and one of the GFP-encoding reporters: let-7 reporter bearing *Cflar*-14, *Mcf2l*-7 or *Utrn*-61 tile downstream of GFP or a negative control without let-7 sites (GFP). Representative smiFISH images (left): *Gfp* RNA, yellow; GFP protein (serving to outline cell borders), magenta; scale bar, 5 μm. Circled are *Gfp* RNA spots quantified using the RS-FISH Fiji plugin^81^. Representative images for *Mcf2l* and *Utrn* reporters are shown in Extended Data Fig. 7. smiFISH quantification (right): the box plots show ratios of the *Gfp* signal in proximal (0–30 μm) versus distal part of neurites (30 μm up to 100 μm); points show ratios for individual neurons. The number of quantified neurons (biological replicates) is indicated below the box plots. *P* values were computed with Welch’s adaptation of *t*-test (two-sided) at 95% confidence interval and adjusted for multiple comparisons using the Benjamini–Hochberg method. Source data

To experimentally confirm the role of the miRNA pathway in mRNA localization, we combined N-zip with *Ago2* depletion using short hairpin RNA (shRNA). RT–qPCR and western blot confirmed a ~70% depletion of *Ago2* (Fig. 5a and Supplementary Table 3). We next analyzed how the enrichment of let-7 targets in neurites changes upon *Ago2* depletion. Compared to N-zip tiles without let-7 sites (zero let-7 sites; Fig. 5b), let-7 targets shifted toward soma, an effect whose degree depended on the number of sites (compare zero with one, two and three let-7 sites).

We then decided to analyze the effect of *Ago2* deletion on the localization of endogenous mRNAs, using mRNA-seq analysis of neurites and soma. Compared with mRNAs that did not contain let-7 sites (gray line, Fig. 5c), let-7 targets shifted their localization toward the soma upon *Ago2* depletion (yellow line: one let-7 7mer seed match; red line: two or more let-7 7mer seed matches).

**Extended Data Fig. 7:**
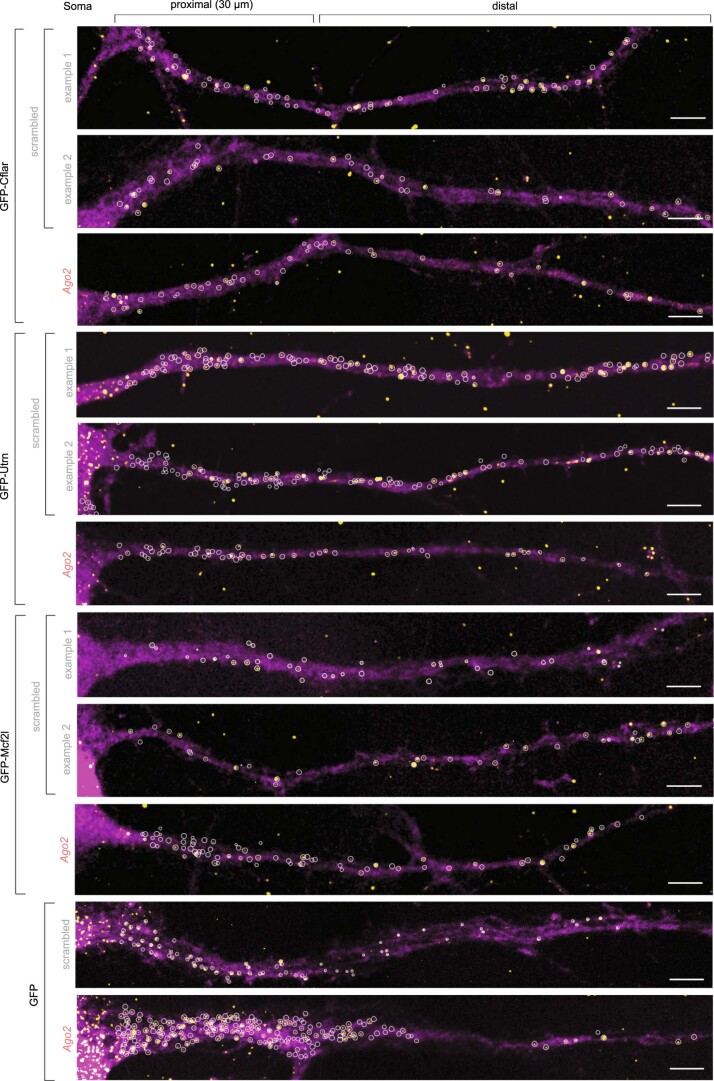
Additional smiFISH images of let-7 reporters in primary cortical neurons. The experimental setup and quantifications are presented in Fig. 5d. Displayed are representative smiFISH images for GFP-Mcf2l and GFP-Utrn reporters and additional to Fig. 5d examples of images showing neurons with different expression levels of GFP-Cflar (examples 1 and 2). *Gfp* RNA, yellow; GFP protein signal (serving to outline cell borders), magenta; scale bar: 5 μm. Circled (white) are fluorescent *Gfp* RNA spots detected and quantified using the RS-FISH Fiji plugin^81^ (see Methods). Each neuron is treated as an independent biological replicate, with 32 to 49 neurons analyzed per each sample. For more details on the number of quantified neurons and statistical analysis of the full dataset, see Fig. 5d.

To validate the effect of *Ago2* depletion on the localization of let-7 targets, we constructed GFP reporters with one of the tiles bearing let-7 binding sites in their 3′ UTR: *Cflar*-14, *Mcf2l*-7 or *Utrn*-61. We used a GFP mRNA without tile sequences (GFP) as a negative control. Upon *Ago2* depletion or treatment with a scrambled shRNA, we transduced these reporters into PCNs and performed single molecule inexpensive FISH (smiFISH)^44^ with GFP-specific probes. Primary cortical cultures are heterogenous and show high variability in reporter expression levels; therefore, to quantify mRNA localization, we compared the signal in the proximal part and in the distal part of neurites within individual neurons (Fig. 5d and Extended Data Fig. 7). Reporter localization in smiFISH recapitulated the results of N-zip. In particular, the incorporation of let-7-bearing tiles in GFP reporters shifted their localization toward distal neurites (Fig. 5d, compare GFP with Cflar, Mcf2l and Utrn, gray boxes). Moreover, *Ago2* depletion reduced localization of let-7 reporters to neurites, supported by significantly higher proximal versus distal signal in *Ago2*-depleted neurons (red) than in scrambled control (gray). Notably, the GFP reporter without let-7 binding sites (‘GFP’) was not significantly affected by *Ago2* depletion.

Given the enrichment of the protein components of the mRNA repression complex in soma (Fig. 4b), we decided to explore if other miRNAs might also affect mRNA localization. We analyzed the frequency of miRNA seeds across differentially localized N-zip reporters and endogenous mRNAs. Consistently with our finding that let-7 is the most abundant miRNA in PCNs (Fig. 4a), the let-7 seed was the only one with statistically significant enrichment in neurite-localized mRNAs (Extended Data Fig. 6c). However, we also detected a modest effect for a few other abundant miRNAs, including miR-154, miR-342 and miR-24 (Extended Data Fig. 6c,d and Supplementary Table 4). These data suggest that other miRNAs have the potential to contribute to mRNA localization, and the ultimate effect may depend on the miRNA expression profiles in individual cell types.

### (AU)_n_-containing mRNAs recruit HSB1L protein to localize to neurites

**Fig. 6:**
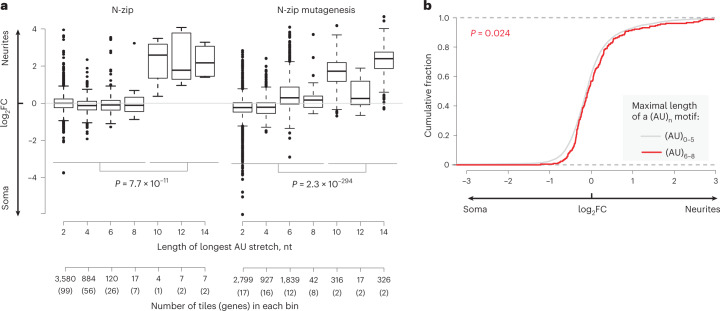
(AU)_n_ motif-containing mRNAs localize to neurites of PCNs. **a**, Box plot showing neurite/soma enrichment (*y*) as a function of the maximal length of the (AU)_n_ stretch length in the tiles in original N-zip (left plot) and mutated N-zip (right plot) mRNA libraries (*x*). The additional *x* axis shows the number of tiles and the number of genes that these tiles were derived from (in parentheses), in which AU stretches of the indicated length were found. *P* values were computed using two-sided Wilcoxon rank-sum test (*n* = 3 independent biological replicates). **b**, Cumulative distribution functions (CDFs) showing fractions of endogenous mRNAs with (AU)_0-5_ (gray) and (AU)_6–8_ stretches (red), as measured by mRNA-seq (*y*), plotted against neurite/soma enrichment. *P* value was computed using two-sided Wilcoxon rank-sum test.

**Extended Data Fig. 8:**
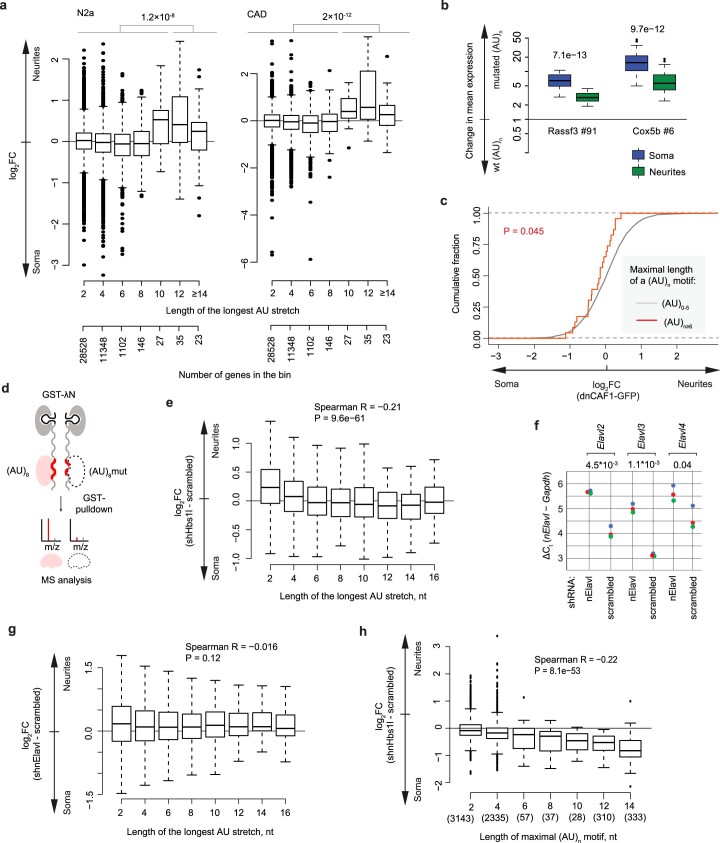
Neurite localization of (AU)_n_ motif is depedent of the motif length and HBS1L protein. (**a**) (AU)_n_ motif is enriched in neurites of neuroblastoma N2a and CAD lines. Boxplot showing neurite/soma enrichment (Y) as a function of (AU)_n_ stretch length (X) in N2a and CAD lines. P-values were computed using two-sided Wilcoxon rank-sum test; n = 3 independent biological replicates. RNA-seq data are from GSE17309838^45^. (**b**) Mutations of (AU)_n_ motifs led to a stronger increase of N-zip mRNA reporter levels in soma. Boxplots showing changes in the normalized expression levels between tiles that contain intact (AU)_n_ motifs (wt (AU)_n_) and tiles in which (AU)_n_ motifs were mutated (mutated (AU)_n_). The data are shown separately for each tile (as indicated on X) and each subcellular compartment (green: neurite, blue: soma); n = 3 independent biological replicates. (**c**) mRNA stabilization with dnCAF1 shift localization of mRNAs with a long AU stretch towards soma. CDFs showing fractions of endogenous mRNAs with (AU)_0-5_ (grey) and (AU)_6-8_ stretches (red), as measured by mRNA-seq (*Y*), plotted against changes in neurite/soma enrichment upon expression of dnCAF1 (*X*). Two-sided Kolmogorov-Smirnov test was used to estimate the significance. (**d**) Scheme for GRNA chromatography in combination with mass spectrometry to identify RBPs bound to (AU)_8_-containing RNA. The (AU)_8_-containing *Rassf3*-91 fragment was tagged with five copies of the boxB sequence, which has a strong affinity to lambda N peptide, and the resulting (AU)_8_-boxB and a negative control with mutated motif (AU)_8_mut-boxB were incubated with mouse brain protein lysates. The complexes formed on (AU)_8_-boxB and (AU)_8_mut-boxB RNAs were then isolated using lambda N-GST fusion protein immobilized on glutathione beads. Protein binders, eluted with RNAse A, were used for further mass spectrometry. (**e**) Changes in mRNA localization upon *Hsb1l* depletion in primary cortical neurons depend on the length of (AU)_n_ motif within mRNA 3’UTR. The data are presented as in (**a**), with changes in neurite/soma enrichment upon *Hbs1l* knockdown plotted on (*Y*). In a linear model of the ratios with the shRNA and the (AU)_n_ motif length as variables, the interaction between the shRNA and the (AU)_n_ was −0.019 (P = 0.00344, computed with Spearman’s correlation test); n = 3 independent biological replicates. (**f**) RT-qPCR showing the efficiency of *Elavl2*, *Elavl3* and *Elavl4* depletion with shRNA in primary cortical neurons. *nElavl* expression levels for *nElavl*-depleted and control scrambled shRNA samples, normalized to *Gapdh* (ΔC_t_), are plotted on (*Y*) as individual biological triplicates (colors dots, n = 3). P-values were computed by two-sided t-test and shown on the plot. (**g**) Changes in mRNA localization upon depletion of nElavl in primary cortical neurons do not correlate with the length of (AU)_n_ motif within mRNA 3’UTR. The data are analyzed and presented as in (**e**); n = 3 independent biological replicates. The interaction in a linear model between the shRNA and the length of the (AU)_n_ motif is not significant (P = 0.8). (**h**) Depletion of *Hbs1l* in primary cortical neurons shifts (AU)_n_-containing N-zip reporters towards soma. Boxplots showing changes in neurite/soma enrichment between *Hbs1l*-depleted and control scrambled samples (Y) as a function of the maximal length of the (AU)_n_ stretch length in the tiles of N-zip mRNA library (X). The number of genes in the bin is shown in parentheses; n = 3 independent biological replicates. Spearman’s correlation coefficient and P-value are shown. In a linear model of the ratios with the shRNA and the (AU)_n_ motif length as variables, the interaction between the shRNA and the (AU)_n_ was -0.07 (P < 10^–16^). Source data

Our N-zip analysis identified (AU)_n_ as a de novo zipcode. We next analyzed how the length of (AU)_n_ affected mRNA localization. We found that N-zip reporters with ≥6 AU repeats were enriched in neurites (Fig. 6a). Similarly, endogenous mRNAs with ≥6 AU repeats in their 3′ UTRs (red line, Fig. 6b) were shifted to neurites compared to transcripts with fewer or no AU repeats (gray line). The effect was weaker for endogenous transcripts than for N-zip reporters, presumably due to other regulatory sequences in the full-length 3′ UTRs. Strikingly, transcripts with ≥5 AU repeats were enriched also in neurites of neuroblastoma lines^45^ (Extended Data Fig. 8a), suggesting that the role of this motif in mRNA localization is conserved in multiple cell types.

We next examined how (AU)_n_ affects transcript levels in soma and neurites. Mutations of (AU)_n_ stabilized N-zip reporters, and this effect was stronger in soma than in neurites (Extended Data Fig. 8b, compare blue and green boxes). As in the case of let-7 targets, expression of dnCAF1 led to stabilization of mRNAs containing ≥6 AU repeats (Extended Data Fig. 6a, compare white and red boxes; *P* < 10^–16^ for both tiles). Moreover, transcripts with a long AU stretch shifted toward soma, when compared with mRNAs carrying short or no AU repeats (Extended Data Fig. 8c). These data suggest that, similarly to let-7 sites, (AU)_n_ mediates enrichment of transcripts in neurites primarily by destabilizing them in soma.

**Fig. 7:**
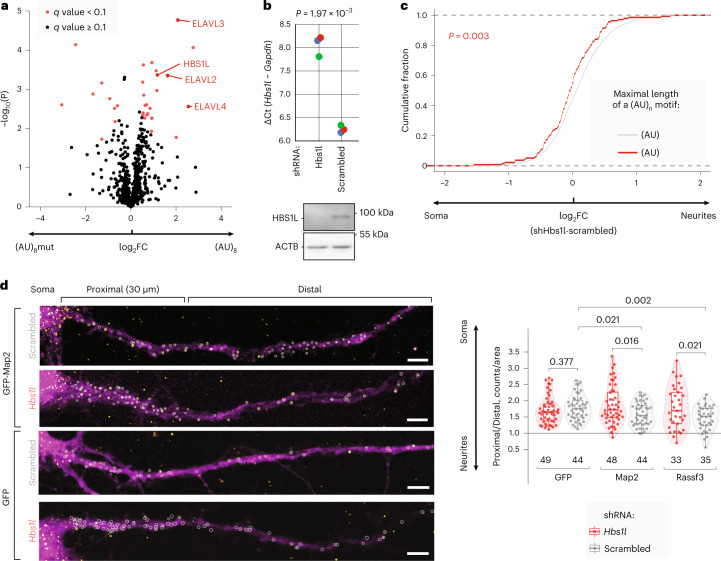
HBS1L binds (AU)_n_ motif to direct mRNA localization to neurites of PCNs. **a**, (AU)_n_ motif binds HBS1L and nELAVL proteins. Volcano plot showing proteins enriched in (AU)_8_-GRNA chromatography. −log_10_ *P* values (*y*, two-sided *t*-test with equal variance) are plotted against log_2_FC of LFQ values between (AU)_8_ and mutant RNA pulldowns (*x*). Proteins with significant *q* value (FDR < 5% using Benjamini–Hochberg correction) are marked in red. **b**, RT–qPCR (top) and western blotting (bottom) show an efficient HBS1L depletion with shRNA in PCNs. *Hbs1l* expression levels for *Hbs1l*-depleted and control scrambled shRNA samples, normalized to *Gapdh* (ΔC_t_), are plotted on *y* as individual biological triplicates (colored dots, *n* = 3). *P* value was computed by two-sided *t*-test. Western blotting showing the expression of HBS1L protein in both samples is provided below the RT–qPCR plot. ACTB was used as a loading control in western blotting. **c**, Depletion of *Hbs1l* in PCNs shifts (AU)_n_-containing mRNAs toward soma. Cumulative distribution functions (CDFs) showing fractions of endogenous mRNAs with no or short (AU)_n_ stretch (*n* = 0–5, gray) and a long (AU)_n_ stretch (*n* > 5, red), as measured by mRNA-seq (*y*), plotted against changes in neurite/soma enrichment upon *Hbs1l* depletion (*x*). *P* value was computed using two-sided Wilcoxon rank-sum test. **d**, smiFISH validates the role of HBS1L in the localization of (AU)_n_-containing reporter. Representative smiFISH images (left) and quantification of smiFISH signal (right) are shown. PCNs were transduced with either *Hbs1l*-targeting (red) or scrambled shRNAs (gray) and one of the GFP-encoding reporters: (AU)_n_ reporter bearing *Map2*-30 or *Rassf3*-91 tile downstream of GFP or a negative control without (AU)_n_ (GFP; Methods). The data were analyzed and presented as in Fig. 5d. The same set of neurons was used as a negative control (GFP reporter with scrambled shRNA) in both smiFISH panels. Representative images for *Rassf3* reporter are shown in Extended Data Fig. 9. Source data

Next, we decided to investigate the RBPs that are recruited by (AU)_n_ and might mediate its localization. Analysis of known RBP motifs suggested that (AU)_n_ could be bound by RBMS1 and RBMS3 (Extended Data Fig. 3a). However, the binding motif for these proteins contains only three AU repeats^46^, which is insufficient for mRNA localization (Fig. 6a). To identify more plausible (AU)_n_ interactors, we used RNA affinity capture^47^ combined with proteomics (Extended Data Fig. 8d). This analysis identified 23 proteins as significantly enriched in complexes formed on (AU)_8_ RNA, compared with a negative control with mutated (AU)_8_ (false discovery rate (FDR) 5%; Fig. 7a and Supplementary Table 6). Among them were HBS1-like protein (HBS1L), critical for cerebellar neurogenesis^48^, and neuronal members of the ELAV-like (nELAVL) family, with the roles in learning and memory^49^.

HBS1L and nELAVLs were reported to have opposite effects on mRNA fate: HBS1L is an mRNA decay factor^50,51^, whereas nELAVL proteins stabilize bound mRNAs by preventing their association with destabilizing proteins^52^. As localization of (AU)_n_-containing mRNAs to neurites is due to their preferential destabilization in soma (Extended Data Fig. 8b,c), HBS1L is a plausible player in this mechanism. Thus, we depleted *Hbs1l* with an shRNA (Fig. 7b) and examined how this depletion affected localization of (AU)_n_-containing transcripts by mRNA-seq of isolated neurites and soma. Remarkably, *Hbs1l* depletion shifted the localization of transcripts with a long (AU)_n_ motif (red line, Fig. 7c, and Extended Data Fig. 8e) toward soma, compared with transcripts with a short or no (AU)_n_ (gray line). In contrast, the same approach applied to nELAVL-depleted neurons (Extended Data Fig. 8f) showed no correlation between the length of (AU)_n_ and changes in mRNA localization (Extended Data Fig. 8g). Depleting *Hbs1l* also changed the localization of N-zip reporters in a manner dependent on (AU)_n_ length (Extended Data Fig. 8h). Thus, these data confirmed the role of HBS1L in localization of (AU)_n_-containing transcripts in PCNs.

**Extended Data Fig. 9:**
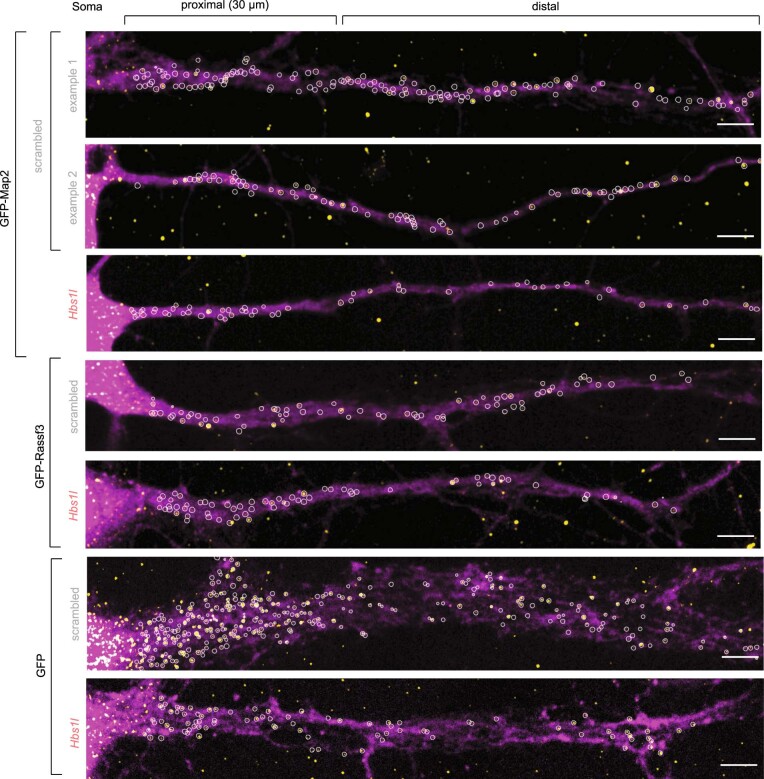
Additional smiFISH images of (AU)_n_ reporters in primary cortical neurons. The experimental setup and quantifications are presented in Fig. 7d. Displayed are representative smiFISH images for GFP-Rassf3 reporter and additional to Fig. 7d examples of images showing neurons with different expression levels of GFP-Map2 (examples 1 and 2). *Gfp* RNA, yellow; GFP protein signal (serving to outline cell borders), magenta; scale bar: 5 μm. Circled (white) are fluorescent *Gfp* RNA spots detected and quantified using the RS-FISH Fiji plugin^81^ (see Methods). Each neuron is treated as an independent biological replicate, with 33 to 49 neurons analyzed per each sample. For more details on the number of quantified neurons and statistical analysis of the full dataset, see Fig. 7d.

To validate the effect of *Hbs1l* depletion on localization of (AU)_n_-containing transcripts by smiFISH^44^, we generated GFP reporters with one of (AU)_n_-carrying tiles in their 3′ UTR (Map2 and Rassf3, Fig. 7d, and Extended Data Fig. 9). As a negative control, we used a GFP construct without any tile in the 3′ UTR (GFP). Quantification of the signal from GFP-specific probes in the proximal and distal part of neurites showed that incorporation of (AU)_n_-bearing tiles in GFP reporters shifted their localization toward distal neurites (Fig. 7d, compare GFP with Map2 and Rassf3, gray boxes); also, *Hbs1l* depletion reduced localization of (AU)_n_ reporters to neurites (Fig. 7d). Indeed, proximal versus distal signal was significantly higher in *Hbs1l*-depleted neurons (red) than in the ones treated with scrambled control (gray). Consistently with the role of (AU)_n_, such an effect was not observed for the control GFP reporter.

## Discussion

Although hundreds to thousands of mRNAs localize to neurites^12–25^, in most cases the mechanisms and biological functions of such localization remain to be understood. Here we present an MRPA^26,53^-based method, N-zip (Fig. 1a), to find zipcodes involved in mRNA localization in neurons. Identifying zipcodes makes it possible to manipulate the localization of specific mRNAs and test their biological roles. Using this method, we analyzed 99 neurite-localized transcripts. For one-third of them, we identified tiles localized to neurites of PCNs (Fig. 1b). Curiously, these transcripts with neurite-localized tiles are associated with local neuronal structures and processes, such as synapse and actin cytoskeleton organization (Extended Data Fig. 3c). Our further analysis of neurite-localized tiles identified several motifs (Extended Data Fig. 3a), two of which— let-7 binding site and (AU)_n_—we characterized in detail and demonstrated their role in mRNA localization in PCNs. We found these two motifs in 15 of the 33 transcripts with a neurite-enriched tile, suggesting that additional mechanisms of localization remain to be characterized in future studies.

mRNA localization can be achieved via different mechanisms. First, mRNAs can be transported along cytoskeletal fibers with the help of motor proteins. In addition, localization can be attained by degrading mRNAs in other regions where they should not be on hand. Notably, whereas the first mechanism generates an mRNA localization pattern by increasing mRNA concentration in one subcellular compartment, the latter achieves the same outcome by decreasing mRNA concentration in another compartment. For example, localization-dependent mRNA degradation has been described for *Hsp83* and *nanos*. Their mRNAs are degraded throughout *Drosophila* eggs via the Smaug-mediated recruitment of deadenylation complex but remain stable at the posterior pole^54,55^. Previous studies reported local processing of miRNAs^56^ and modification of the components of the miRNA pathway in response to synaptic activity^57–59^. However, to our knowledge, localization-dependent degradation of mRNAs by miRNAs has not been previously described. Given the known role of miRNAs in mRNA degradation (reviewed in ref. ^40^), it seems highly likely that this could be used as a mechanism to establish pools of specific mRNAs in some areas of the cell by degrading them in others. N-zip identified the binding site for an miRNA—let-7—as a zipcode (Fig. 1b2). Let-7 is the most abundant miRNA in the mammalian brain^56,60,61^ (Fig. 4a) and is involved in neuronal differentiation^62^, regeneration^63,64^ and synapse formation^65,66^. Our results point to a new neuronal function for let-7 in mediating mRNA localization.

Relatively moderate effects of miRNAs on their targets (~1.3-fold downregulation on average^67,68^) may enable many mRNA molecules to escape degradation by miRNAs in soma and localize to neurites. Intriguingly, let-7 was equally abundant in the neurites and soma of PCNs (Fig. 4a), raising the question about the mechanism underpinning the higher let-7 activity in soma. One explanation came from our proteomic analysis, which has shown that protein components of miRNA machinery are enriched in soma (Fig. 4c), leading to lower levels of let-7 targets in soma (Fig. 4b).

It remains to be understood how such mRNAs, regulated via differential stability, are transported into neurites. In long and thin neurites, diffusion seems unlikely to suffice in transporting mRNAs at hundreds of micrometers. It is tempting to speculate that they may be transported with the help of motor proteins. In the simplistic view of motor-dependent transport, RBPs recognize specific localization elements in mRNA and tether them to motor proteins for transport (reviewed in ref. ^1^). However, in vivo, this process appears to be more complex, involving the formation of higher-order messenger ribonucleoprotein (mRNP) transport granules. Such granules can be formed through phase separation and contain numerous mRNAs and RBPs co-transported with a limited set of motor proteins (reviewed in ref. ^69^). Non-selective inclusion of mRNA in such granules may facilitate their transport to neurites, whereas selective mRNA degradation would generate an mRNA gradient across the cell.

We identified (AU)_n_ as another de novo zipcode in PCNs. (AU)_n_ is found in important neuritically enriched mRNAs, including *Map2* and *Shank3* (Supplementary Table 1 and Extended Data Fig. 4b). RNA affinity capture showed that (AU)_n_ is bound by an mRNA decay factor HBS1L and nELAVL proteins (Fig. 7a), both having important functions in neuronal development^48,49^. Based on RIP-Chip and SELEX experiments^70^, ELAVL proteins bind AU-rich elements (AREs), including UUUAUUU and its variations. AREs bear some resemblance to (AU)_n_, which may explain detection of nELAVLs among (AU)_n_ interactors. However, nELAVLs stabilize their bound mRNAs (reviewed in ref. ^52^), whereas our analysis showed that localization of (AU)_n_-containing transcripts is mediated by selective mRNA destabilization (Extended Data Fig. 8b,c). Indeed, depletion of nELAVLs did not decrease neurite enrichment of (AU)_n_-containing transcripts (Extended Data Fig. 8f,g).

Our depletion experiments showed that HBS1L mediated localization of (AU)_n_-containing mRNAs (Fig. 7b–d and Extended Data Fig. 8e,h). HBS1L is involved in mRNA quality control pathways, including No-Go and Nonstop decay, that degrade mRNAs with stalls in translation elongation (reviewed in ref. ^71^). HBS1L belongs to the GTPases family and is homologous to translation elongation and termination factors eEF1 and eRF3. In this study, we uncovered the role of HBS1L in the localization of (AU)_n_-containing transcripts via selective degradation in soma. It remains to be understood how it is recruited to (AU)_n_ to trigger mRNA degradation.

Parallel studies^45,72^ describe similar approaches to identifying zipcodes in neuroblastoma cell lines. We detected no overlap with the results of Arora et al.^72^, most probably because their MRPA library included only eight neurite-localized transcripts. Our analysis of the Mikl et al.^45^ data showed that the (AU)_n_ motif is linked to mRNA localization not only in cortical neurons but also in neuroblastoma lines (Extended Data Fig. 8a). Moreover, binding motifs for CELF/BRUNOL and PCBP family members, identified among the binders of the neurite-localized synthetic sequence by Mikl et al.^45^, were also overrepresented in neurite-localized N-zip tiles (Extended Data Fig. 3a). Both CELF/BRUNOL and PCBP protein families are broadly involved in RNA metabolism. Curiously, CELF/BRUNOL is involved in localized translation in *Drosophila* oocyte^73,74^. In contrast, let-7 binding sites function as zipcodes in PCNs (Fig. 3c,d) but not in neuroblastoma lines. This is likely due to differences in let-7 expression levels between primary neurons and neuronal cell lines^75^. This example illustrates the merits of using primary cells to identify functional elements with biological roles in vivo.

**Extended Data Fig. 10:**
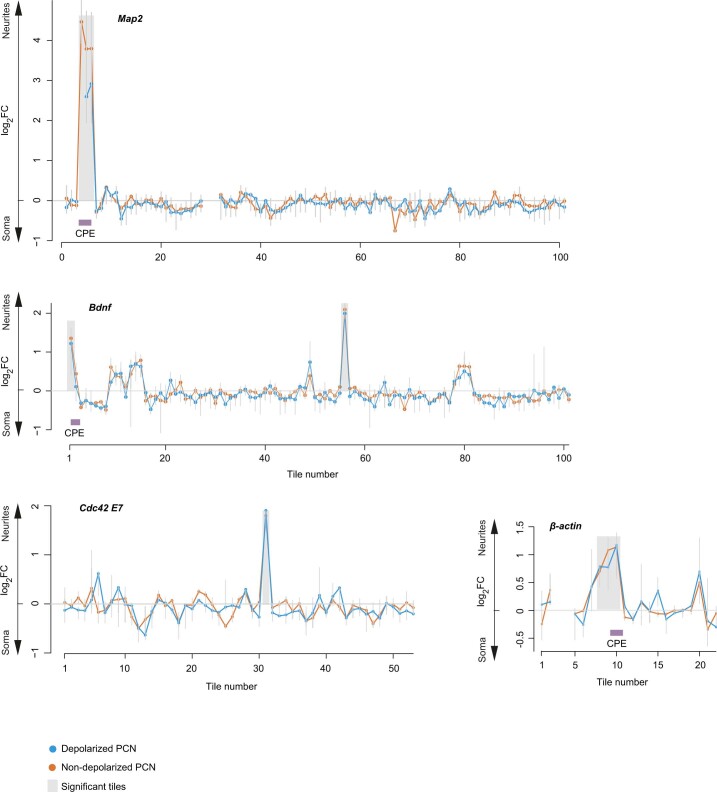
N-zip identifies and refines known zipcodes in primary cortical neurons. Specific examples of identified tiled fragments that mediate localization to neurites. Enrichment of a given tile (log_2_-transformed fold change) between subcellular compartments (*Y*) is plotted against tiled fragment number (*X*). Average neurite/soma ratios for depolarized (blue) and non-depolarized neurons (orange) are plotted. Error bars represent the lowest and the highest measurement for each tile. Shaded regions indicate tiles with significant enrichment (P < 0.05) in neurites by more than 2-fold. P-values were computed with DESeq2 two-sided Wald test. In the case of adjacent tiles with significant enrichment, the neighboring tiles with lower than 2-fold enrichment are also shaded. CPE is defined as a stretch of 6 bases including 5 Us. The gene name is shown above each plot.

N-zip has also identified and refined several previously known zipcodes. For example, the CPE was reported to facilitate mRNA transport to dendrites and play a role in *Map2* (ref. ^8^) and *Bdnf* (ref. ^9^) localization. In line with that, CPE-containing tiles in *Map2* (tiles 4–6) and *Bdnf* (tile 1) were neurite-localized in N-zip (Extended Data Fig. 10). As a group, tiles containing CPE in the last 30 bases tended to become more neurite-enriched upon depolarization (*P* = 7.7 × 10^–15^). In contrast, no effect was observed when considering the whole tile (*P* = 0.41), suggesting that CPE is more functional when located closer to the polyadenylation signal, consistent with previous literature^76^. Moreover, we identified the second localization element in *Bdnf* (tile 56; Extended Data Fig. 10) within the region previously shown to contribute to dendritic localization of *Bdnf*^77^. We also found that tile 31 in the neuritically localized isoform of *Cdc42* (ref. ^25^) is sufficient for localization.

Curiously, depolarization activated localization of tiles mapping to transcripts with essential functions in neurites, including mRNAs encoding ribosomal proteins (Extended Data Fig. 2). Localization of these transcripts to neurites is conserved across multiple types of neurons^31^, hinting to their importance in local translation. Indeed, newly translated ribosomal proteins provide for ribosome maintenance in neurites^78,79^. Localization of mRNAs encoding for different ribosomal proteins may also generate specialized pools of ribosomes translating local transcriptome. In addition, depolarization stimulated localization of a tile from *Adcy1*, encoding an enzyme that catalyzes the formation of the signaling molecule cAMP and plays an essential role in memory and learning^37^.

It should be noted that N-zip is limited to the detection of relatively short (≤150-nt) zipcodes. For example, the zipcode of *Arc*, containing a 350-nt region^11^, is too long to be mapped by N-zip. Zipcodes consisting of multiple motifs residing in different 3′ UTR parts and those dependent on splicing are also not detectable in our current implementation of N-zip. A further limitation is that we are studying the activity of zipcodes in a fixed backbone, and different backbones, along with their length, GC content and splicing status, can have variable effects on the activity of individual tiles^53^. Our mutagenesis approach can detect zipcodes that are disrupted by mutations of single nucleotides or longer kmers, but it is limited in the detection of redundantly acting zipcodes. Lastly, the activity of individual zipcodes can depend on the developmental stage and neuronal activity.

Let-7 binding sites and (AU)_n_ motifs help localize both exogenously introduced N-zip reporters and endogenous mRNAs, but the effect is stronger for N-zip reporters. The likely reason is that the full-length 3′ UTRs of endogenous mRNAs are longer and potentially carry additional regulatory elements that may exercise a finer control over mRNA localization. It can also affect the distance between the elements and the poly(A) tails. The ultimate localization of these mRNAs is a combinatorial result based on multiple regulatory sequences. In support of this, a single 3′ UTR often harbors sequences that promote and inhibit neurite enrichment (Fig. 1b). In summary, N-zip allows decoding the combinatorial effects that regulate mRNA localization by zooming in on shorter sequences with specific roles in localization. This is a crucial step toward unraveling how a finite number of patterns produce many types of polarized cells and help them adapt to challenges and changes.

## Methods

Our research complies with all relevant ethical regulations and has been approved by the Max Delbrück Center (MDC) for Molecular Medicine and the German regulation authority: das Landesamt für Gesundheit und Soziales (LAGeSo).

### Experimental models

The HeLa-rtTA cell line used for luciferase reporter assay was obtained from Kai Schoenig (ZI Mannheim)^82^, and the 293T cell line used for lentivirus production was obtained from the MDC. Male and female *Mus musculus* embryos (embryonic day 14 (E14)) or neonatal pups (P0) of the C57BL/6J strain, obtained from the MDC mouse facility, were used to prepare cortical neuron cultures. The number of animals dissected for each experiment was defined by the number of neurons required for plating (see ‘PCN culture and lentiviral transduction’ subsection) and calculated based on the expected yield of 5 × 10^6^ neurons per pup.

### PCN culture and lentiviral transduction

PCN culture, separation on soma and neurites and lentivirus preparation were done as described previously^25^. In short, cortical neurons were isolated from E14 or P0 pups and co-cultured with astrocytes^83^. For separation on neurites and soma, 1 × 10^5^ cells per cm^2^ neurons were plated on double-coated (poly-d-lysine and laminin) cell inserts (Millicell six-well PISP30R48, Millipore). Soma was dissociated from the top of inserts by intensive washes with cold PBS and spinning at 5,000*g* and 4 °C for 1.5 minutes. For neurites isolation, cotton swabs were used to remove the remaining soma from the top of the insert, and the membrane with attached neurites was used for protein or RNA isolation. For protein lysates, neurites and soma were lysed in 8 M urea and 0.1 M Tris-HCl pH 7.5. TRIzol reagent (Thermo Fisher Scientific) was used for RNA isolation.

To prepare lentiviral particles, 293T cells growing in 10-cm dishes were transfected using polyethylenimine (PEI) with 10 µg of the envelope (Addgene, 12259), packaging (Addgene, 12260) and transfer plasmids in the ratio 1:1:2. The next day, the medium was exchanged to 10 ml of DMEM without FBS. Seventy-two hours after transfection, the lentivirus-containing medium was cleared from cell debris by centrifugation at 500*g* for 5 minutes and concentrated at 4 °C for 4–24 hours using 3 volumes of Lenti-X concentrator (631232, Takara Bio). Viral particles were collected by centrifugation at 1,500*g* and 4 °C for 45 minutes and resuspended in 200 µl of cold PBS. Virus was applied on cortical neurons between days in vitro 3 (DIV3) and DIV6, and cells were collected at DIV9. For N-zip experiments, 70 µl of the concentrated virus was added per 10^6^ PCNs growing on a Millicell cell insert (six-well) at DIV5. Preparation of the shRNA depletion samples for RNA-seq of endogenous RNAs was performed similarly, with 30 µl of the concentrated virus transduced. For shRNA depletion experiments combined with N-zip libraries, 30 µl of shRNA virus was added to cells at DIV3; the medium was changed at DIV5; and 70 µl of viral N-zip library was added at DIV6.

For depolarization experiments, P0 PCNs were treated as described previously^36^. In brief, on DIV8, the cells were treated for 16 hours with 50 µM APV and 10 µM CNQX to block NMDA and AMPA receptors. Neurons were then stimulated with a final concentration of 55 mM KCl using 3× KCl depolarization solution (170 mM KCl, 10 mM HEPES pH 7.4, 1 mM MgCl_2_ and 2 mM CaCl_2_). RNA for RT–qPCR and N-zip library preparation was collected from cells before depolarization (control samples) and after sustained 3-hour depolarization. The experiment was performed in biological triplicates.

### Luciferase reporter assays

Human HeLa-rtTA cells expressing reverse tetracycline-controlled transactivator^82^ were grown in DMEM with GlutaMAX supplement (DMEM + GlutaMAX, Gibco) with 10% FBS and used in luciferase reporter assay. Transfections were done in 96-well plates with PEI using a 1:3 ratio of DNA:PEI. Cells were transfected with 1–3 ng of FL/RL doxycycline-inducible let-7 reporter per well. Increasing amounts of GFP-let-7 sponge (40, 60, 80, 100 and 125 ng per well) were co-transfected, where indicated. GFP-encoding plasmid was used as a filler, to top up each transfection to the same total amount of DNA. Expression of luciferase reporters was induced with doxycycline (1 µg ml^−1^), and cells were lysed 24 hours after transfection. Luciferase activities were measured with a homemade luciferase reporter assay system as described previously^84^. More specifically, 45 µl of FLuc reagent (75 mM HEPES pH 8.0, 0.1 mM EDTA, 4 mM MgSO_4_, 530 µM ATP, 270 µM coenzyme A, 470 µM DTT and 470 µM luciferin) and 45 µl of RLuc reagent (2.2 mM Na_2_EDTA, 220 mM K_3_PO_4_ pH 5.1, 0.44 mg ml^−1^ of BSA, 1.1 M NaCl, 1.3 mM NaN_3_ and 0.6 µg ml^−1^ of coelenterazine) reagents per sample were used to measure luciferase activities.

### DNA constructs

FL/RL-hmga2-wt and FL/RL-hmga2-mut were previously described^42,85^. For other let-7 reporters, we modified the same backbone with bi-directional promoter for simultaneous expression of two genes, pSF2.GFPLuc^86^. First, *Renilla* luciferase (RL) was PCR-amplified and cloned into EcoRI/NotI-cut pSF2.GFPLuc, to substitute GFP with RL and produce pSF2-FL/RL. Next, a fragment containing the polyadenylation signal was cloned into NotI site downstream of RL, to generate pSF2-FL/RL-pA. Finally, synthetic oligos corresponding to Cflar-14, Mcf2l-7 and Utrn-61 tiles (Supplementary Table 1) or their mutated versions (CTACCTC → CCATCCC and CTACCTC → GATGGAG) were annealed and cloned between AgeI and NotI sites of pSF2-FL/RL-pA.

To generate a GFP-encoding plasmid, GFP open reading frame (ORF) was PCR-amplified and cloned between AgeI and EcoRI sites of the lentiviral vector with synapsin I promoter (Addgene, 20945). During this cloning, the AgeI site was destroyed, and a new AgeI was introduced on a PCR primer downstream of GFP. The resulting pLenti-GFP construct was used to produce pLenti-GFP-Map2, pLenti-GFP-Cflar, pLenti-GFP-Rassf3, pLenti-GFP-Mcf2l, pLenti-GFP-Utrn and pooled N-zip lentiviral libraries. To generate pLenti-GFP reporters with N-zip tiles in their 3′ UTR, the corresponding annealed oligonucleotides (Supplementary Table 7) were cloned between AgeI and EcoRI sites downstream of GFP.

To produce (AU)_8_-boxB and (AU)_8_mut-boxB constructs for GRNA chromatography, synthetic oligos corresponding to *Rassf3-91* tile or its mutated version (ATATATATATATATAT → GTACATACATGTACAT) were annealed and cloned between KpnI and NheI sites of pBS-Luc-boxB^87^.

pLKO-shAgo2 and pLKO-shHbs1l were generated by cloning of annealed oligonucleotides (Supplementary Table 7; shAgo2-fw and shAgo2-rev, shHbs1l-fw and shHbs1l-rev, correspondingly) into AgeI and EcoRI-cut pLKO1-puro vector (Addgene, 8453).

### Western blotting

Next, 5–10 µg of total protein was separated on a 10% Laemmli PAAG. Proteins were transferred to the PVDF membrane and analyzed by western blotting. The following primary antibodies were used: rabbit anti-histone H3 1:5,000 (ab1791, Abcam), mouse anti-actin 1:4,000 (Sigma-Aldrich, A2228), mouse anti-neurofilament SMI312 1:10,000 (837904, BioLegend), rabbit anti-Hbs1l 1:500 (H00010724-PW1, Abnova) and mouse anti-Ago2/eIF2C2 1:500 (H00027161-M01, Abnova). Western blot images shown in Figs. 5a and 7b and Extended Data Fig. 1b have been cropped for presentation. Full-size images are presented in Source Data.

### smiFISH probe design and preparation

To assay RNA localization of GFP reporter constructs, complementary 18–20-nt DNA probes against the ORF of GFP (24 probes) were designed using the Biosearch Technologies Stellaris RNA FISH probe designer tool (https://biosearchtech.com). All probe sequences are provided in Supplementary Table 7. The reverse complement of the X FLAP^44^ sequence was added to the 5′ end of each probe: CCTCCTAAGTTTCGAGCTGGACTCAGTG. The X FLAP oligo (CACTGAGTCCAGCTCGAAACTTAGGAGG), 5′ and 3′ end-labeled with Quasar 570, was synthesized by Biosearch Technologies. X FLAP was hybridized with the probe set using the following conditions: 2 μl of the probe set (40 pmol in total), 0.5 μl of 100 μM X FLAP, 1 μl of 10× NEB 3 buffer and 6.5 μl of water were mixed and annealed in a thermal cycler as described previously^44^: 85 °C for 3 minutes, 65 °C for 3 minutes, 25 °C for 5 minutes and 4 °C hold. Annealed probes were stored at −20 °C.

### smiFISH imaging

smiFISH was performed with *GFP* probes on mouse PCNs (P0 and DIV9) cultured on 15-mm glass coverslips as described previously^44^, with some modifications. The media was aspirated, and cells were washed with 1× PBS. Cells were fixed with 4% paraformaldehyde (PFA) for 20 minutes at room temperature and then rinsed twice with 1× PBS. Permeabilization was done with 70% ethanol at 4 °C overnight. The cells were washed with 15% formamide freshly prepared in 1× SSC buffer for 15 minutes at room temperature. Then, 50 μl of hybridization mix (1 μl of FLAP hybridized probes in 2× SSC, 10% formamide and 10% w/v dextran sulfate) was added to each coverslip and incubated overnight at 37 °C in a humid chamber. The cells were washed twice for 30 minutes with freshly prepared 15% formamide/1× SSC at 37 °C in the dark. During the second wash, DAPI nuclear stain was added (5 ng μl^−1^). The cells were washed twice more in 2× SSC, and the coverslips were mounted on a glass slide with 10 μl of ProLong Glass mounting medium (Thermo Fisher Scientific, P36980) without DAPI. Then, 3 × 3 tiled z-stack images were acquired using a Dragonfly 200 spinning disc confocal microscope with a ×63 oil objective and stitched using Fusion acquisition software. Consistent laser intensity and exposure times were used across samples to detect DAPI, EGFP protein and Quasar 570 (EGFP RNA).

### smiFISH image analysis

The images were analyzed using radial symmetry-FISH (RS-FISH)^81^. In brief, maximum projections were performed using Fiji (ImageJ)^88^ for EGFP and used to generate a mask for soma and neurites using ilastik^89^ and manual curation using Fiji to generate masks for proximal neurite (up to 30 μm from the cell body/soma) and distal neurite (30 μm up to a maximum of 100 μm). The RNA foci were detected and quantified using the RS-FISH Fiji plugin as described in Bahry et al.^81^. The detections were subsequently filtered using either the distal neurite or proximal neurite masks using the mask filtering tool in RS-FISH. The RNA detections were normalized by area in their region. Scripts for smiFISH image analysis can be found at https://github.com/LauraBreimann/smFISH_neuron_analysis.

### RNA extraction and qRT–PCR

For RT–qPCR analysis, RNA was isolated with TRIzol (Thermo Fisher Scientific), treated with RQ1 DNase I and reverse-transcribed using the Maxima first-strand cDNA synthesis kit (Thermo Fisher Scientific). *Ago2*, *Hbs1l* and *Gapdh* were quantified using sensiFAST SYBR No ROX qPCR kit (Bioline). *Homer and Snord15b* were used to estimate the efficiency of soma/neurite separation. See Supplementary Table 7 for primer sequences. Relative expression levels were calculated using the ΔΔC_t_ method with *Gapdh* as a reference gene.

### In vitro transcription, GRNA chromatography and mass spectrometry

(AU)_8_-boxB and (AU)_8_mut-boxB RNAs were generated using a T3 MEGAscript in vitro transcription kit (Thermo Fisher Scientific, AM1338) according to the manufacturer’s recommendations. The template plasmids were linearized with HindIII. RNA was purified using Agencourt RNAClean XP beads (Beckman Coulter, A63987).

GRNA chromatography was performed as described previously^87^ with the following modifications. First, 30 µg ml^−1^ of GST-lambda N fusion peptide was immobilized on 20 µl of a 50% slurry of Glutathione Sepharose 4B (Amersham, 17075601) in binding buffer (BB: 20 mM Tris-HCl pH 7.5, 150 mM NaCl, 10% glycerol, 0.05% NP-40 and 0.4 mM) by incubating on an orbital rocker for 30 minutes at room temperature. Beads were washed twice in 1 ml of BB and incubated with 25 pmol of RNA ((AU)8-boxB and (AU)8mut-boxB) in 200 µl of BB for 1 hour at 4 °C. The beads were washed twice with 1 ml of BB and incubated with 3 mg of protein lysate prepared from P0 mouse brain (lysis buffer: BB with 0.5% NP-40) for 2 hours at 4 °C. The beads were washed three times with 1 ml of BB, and bound proteins were eluted with 0.15 µg of RNAse A in 60 µl of BB without NP-40 for 30 minutes at 30 °C on an orbital shaker. Eluates were supplemented with 70 µl of 2.5 M NaOAC pH 5.0, 1 µl of GlycoBlue (Ambion) and absolute EtOH up to 2 ml and incubated at 4 °C overnight. Proteins were recovered by centrifugation at 18,000*g* and 4 °C for 30 minutes. Total protein lysates from neurites and soma of PCNs were prepared as previously described^24^. Eluates and total lysates were subjected to in-solution digest using trypsin, and desalted peptides were subjected to liquid chromatography–tandem mass spectrometry (LC–MS/MS) using a Q Exactive HF-X mass spectrometer coupled to an Easy nLC 1200 system (Thermo Fisher Scientific). MS data were processed with Max Quant software (1.6.3.4) with peptide FDR cutoff at 1%. The resulting text files were filtered to exclude reverse database hits, potential contaminants and proteins identified only by site. For eluate samples, label-free quantification (LFQ) intensity values were filtered for ‘minimum value of 3’ in at least one group. Missing values were imputed with random noise simulating the detection limit of the mass spectrometer. Differential proteins were defined using two-sample Studentʼs *t*-test and FDR-based significance cutoff. The DEP R package (version 1.6.1)^90^ was used to analyze iBAQ protein intensity values from total proteome data. Only proteins detected in at least half (three out of six) of samples and not marked as potential contaminant or reverse sequence were retained for analysis. Missing values were imputed using the ‘MinProb’ algorithm (random draws from a Gaussian distribution) with standard settings, and values from each compartment were then normalized to the median GAPDH intensity. Enrichment between compartments was calculated using a generalized linear model (limma), and *P* values were FDR-corrected with the Benjamini–Hochberg method.

### mRNA-seq and total RNA-seq libraries preparation

mRNA-seq libraries were prepared with TruSeq Stranded mRNA library preparation kit (Illumina, 2002059) according to the manufacturer’s recommendations. mRNA-seq was done in biological triplicates, using 100 ng of total RNA from neurites or soma per sample. RNA was supplemented with ERCC spike-ins (Thermo Fisher Scientific, 4456740) according to the manufacturer’s recommendations. Based on the spike-ins, we estimate that ~4% of mRNA per PCN is contained in neurites and the rest in soma. SLAM-seq library preparation and data analysis are described elsewhere (E-MTAB-11572 and E-MTAB-11575, https://github.com/melonheader/Stability). Total RNA-seq libraries (Extended Data Fig. 1) were prepared using TruSeq Stranded total RNA library preparation kit (20020596, Illumina). Libraries were pooled and sequenced on Illumina NextSeq 500 or NovaSeq 6000 system with a single-end 75-cycle or 150-cycle run.

### N-zip libraries preparation

To generate a pooled lentiviral library expressing fragments tiled across 3′ UTRs of neurite-localized transcripts, a pool of the corresponding oligos (Supplementary Tables 1 and 2) flanked by adapter sequences (TTCGATATCCGCATGCTAGC-tile-GATCGGAAGAGCACACGTCT) was synthesized (Twist Bioscience or Agilent Technologies). Fragments were PCR-amplified (see Supplementary Table 7 for primer sequences) and cloned via Gibson assembly into AgeI-cut pLenti-GFP downstream of GFP, using Endura ElectroCompetent Cells (Lucigen, 60242-1). Resulting pooled DNA libraries were used to produce lentiviral particles and infect PCNs.

RNA, isolated from neurites and soma of PCNs as previously described^25^, was used to prepare N-zip libraries. In short, RNA was reverse-transcribed into cDNA with a primer complementary to the 3′ adapter flanking the tiles. Unique molecular identifiers (UMIs) were introduced during the second-strand synthesis with a pool of primers complementary to the 5′ adapter flanking the tiles. Residual primers were removed with an ExoI/rSAP mix. The resulting double-stranded DNA (dsDNA) was purified with AMPure beads, and the N-zip tiles were PCR-amplified and barcoded. Libraries were pooled and sequenced on Illumina NextSeq 500 or NovaSeq 6000 system with a single-end 75-cycle or 150-cycle run.

### Analysis of published RNA-seq datasets

Several transcriptome datasets from compartments of primary neurons were acquired from published sources: raw datasets were downloaded from the Gene Expression Omnibus (GEO) database (GSE67828 (ref. ^20^), GSE66230 (ref. ^22^) and GSE51572 (ref. ^29^)) and analyzed using the PiGx RNA-seq pipeline^91^ (version 0.0.3) with default settings using the Ensembl mouse genome assembly (GRCm38.p6, release 91); alternatively, counts were taken from supplementary tables of studies that either did not deposit raw data^28^ or did not use a standard RNA-seq approach^21,30^. Differential expression analysis between neurite and soma compartments was performed using DESeq2 (ref. ^92^). Additionally, counts were normalized to transcripts per million (TPM) and averaged for neurite and soma compartment within each dataset.

### Analysis of PCN RNA-seq data

RNA-seq data from our PCNs were analyzed with the PiGx pipeline in the same way as published datasets. Because genomic and intronic reads were detected in the stranded library, a restricted set of genes was chosen for analysis: strand-specific counts (sense and antisense) as well as intron counts were generated using a custom ht-seq-based^93^ Python script. Only genes with strong exon/intron and sense-strand/antisense-strand ratios (log_2_(exon/intron) > 2.5 and log_2_(sense/antisense) > 2) were used for further analysis.

### Selection of zipcode candidate 3′ UTRs

To select sequences for the first N-zip library, only genes with significant enrichment (adjusted *P* < 0.05) in the analyzed primary neuronal datasets were considered as candidates to generate a list of genes with reliable neurite localization. This selection was further restricted to genes for which an enrichment value could be calculated in our PCN system (log_2_FC not NA). Then, genes with (1) a significant enrichment in at least four datasets; (2) median log_2_FC > 1; and (3) either mean log_2_FC > 1 or a positive log_2_FC value in all datasets were chosen. Additionally, genes with a significant enrichment value in at least five datasets and either median log_2_FC > 1 or mean log_2_FC > 1 or a positive log_2_FC value in all datasets were chosen as well.

This initial unbiased set of genes was then manually refined by (1) excluding genes encoded by the mitochondrial genome as well as some genes with the annotated nuclear or mitochondrial function (*Pola1*, *Ezh2*, *Smc4*, *Cenpb*, *Pink1* and *Ncl*); (2) adding genes with a known zipcode or neurite localization sequence (*Camk2a*^94^, *Actb*^5^, *Bdnf*^9^, *Arc*^11,95^, *Cdc42* (ref. ^25^), *Map2* (ref. ^96^) and *Bc1* (ref. ^97^); (3) adding genes that showed localization in non-primary^31^ and in-house datasets as well as our PCN and fewer other primary datasets (*Rab13*, *Net1*, *Hmgn5*, *2410006H16Rik*, *Pfdn5*, *Tagln2*, *Pfdn1* and *Cryab*); and (4) restricting the genes encoding for ribosomal proteins and translation factors to a smaller subset with sufficiently large 3′ UTRs (*Rplp2*, *Rpl12*, *Rpl39*, *Rpl37*, *Rpl14*, *Rps28*, *Rpsa*, *Rps24*, *Rps23*, *Rps18*, *Eef1b2*, *Eef1a1* and *Eef1g*).

### Design of the 3′ UTR tile library

The 3′ UTR sequences for all transcript isoforms of the chosen genes were downloaded via Ensembl biomaRt. For each gene, the 3′ UTR sequences fully contained in another isoform were removed, leaving only the longest non-overlapping 3′ UTR sequences. For all genes with multiple unique 3′ UTR sequences, we manually decided which isoform sequence(s) to include in the final set of sequences, based on annotation and PCN genome browser tracks of the corresponding genes. For *Cflar* and *Cdc42*, both alternative 3′ UTRs were included (and named *Cflar*-1 and *Cflar*-2, respectively, for *Cdc42*). For *Hdac5* and *Arhgap11a*, the different but overlapping isoforms were manually merged into one sequence. This resulted in a final list of 99 3′ UTR sequences.

Each of these sequences was then cut into overlapping tiles covering the entire sequence. For sequences smaller than 500 nt, tiles with 75-nt size and 15-nt offset were designed. For sequences larger than 500 nt, tiles with 100-nt size with 25-nt offset were generated. In both cases, any remaining sequence was added to the last tile while keeping the maximum tile size below 80 nt or 110 nt, correspondingly. In addition, five control tiles with scrambled sequences were generated from the first tile of *Camk2a*, *Actb* and *Bc1* each. The final set of 4,813 tiles was ordered, including 3′ and 5′ adapter sequences (3′: TTCGATATCCGCATGCTAGC; 5′: GATCGGAAGAGCACACGTCT). The full sequences are provided in Supplementary Table 1.

### N-zip data analysis

The sequenced reads were used to count individual library tiles. We considered only R1 reads that contained the TTCGATATCCGCATGCTAGC adaptor sequence and extracted the UMI sequence preceding the adaptor. Each read was then matched to the sequences in the library, without allowing insertions or deletions. The output from this step was a table of counts of reads mapping to each library sequence. The N-zip libraries had 4.8 million mapped reads on average, and the mutagenized N-zip had 1.9 million mapped reads on average. Only fragments with at least 20 reads on average were used in subsequent analysis. In the N-zip library, 4,745 tiles had at least three samples with at least 20 reads (98.5%). The mutagenized N-zip library had 6,266 tiles, and 5,679 (90.6%) had at least three samples with at least 20 reads. The reads were normalized to the total number of mapped reads. Counts within each compartment were highly correlated between replicates (Spearman R > 0.85). The normalized counts were used to compute neurite/soma ratios after adding a pseudocount of 0.5. Statistical significance for each tile was estimated with DESeq2ʼs Wald test (default) and corrected for multiple testing using the Benjamini–Hochberg procedure. Ratios for groups of tiles were compared using two-sided Wilcoxon rank-sum test. The code for N-zip data analysis is available at https://github.com/IgorUlitsky/MPRNA.

### Analysis of miRNA binding sites and (AU)_n_ motif

Analysis of miRNA binding sites and (AU)_n_ motif enrichment was performed on N-zip libraries and mRNA-seq libraries. Tile sequences and sequences of 3′ UTRs of protein-coding genes annotated in GENCODE were analyzed using the R ‘stringr’ package (version 1.4.0) for matches to the let-7 seed sequence and for the maximal match to the (AU)_n_ motif. For obtaining neurites/soma ratios in mRNA-seq data, reads were mapped to the transcriptome using Salmon, and transcript abundance and enrichment was calculated using tximport and DESeq2 workflow^92^. Only the most abundant isoforms, with total TPM of at least 10 in the averaged soma and neurites samples were considered in all further analyses. For analysis of the shRNA-treated cells, we used RSEM and GENCODE vM21 to obtain isoform-specific expression levels quantified as TPM. These were used to compute log_2_-transformed ratios between the averages of the neurite and the soma samples using a pseudocount of 1.

### Design of mutation tile library

From the first N-zip library, a subset of neurite-enriched tiles was chosen for extensive mutagenesis. In case of overlapping tiles forming one peak, the central tile was selected. More specifically, from the tiles with significant (adjusted *P* < 0.1) and high (mean log_2_FC > 1) enrichment in neurites, we chose the following 16 tiles: *Ndufa2*, tiles 11 + 12 (for 75-nt tiles, two tiles were combined); *Camk2n1*, tile 12; *Msn*, tile 48; *Golim4*, tile 56; *Cdc42*_2, tile 31; *Bdnf*, tile 56; *Map2*, tile 5; *Cflar*_2, tile 52; *Rassf3*, tile 91; *Mcf2l*, tile 7; *Cflar*_1, tile 14; *Utrn*, tile 61; *Cald1*, tile 58; *Rps23*, tiles 11 + 12; *Cox5b*, tiles 6 + 7; and *Kif1c*, tile 80. For each of these tiles, we generated all possible single base substitutions as well as sets of A ⇄ T and C ⇄ G base transitions in 2mer, 5mer and 10mer stretches that together cover each tile. Additionally, the wild-type and three scrambled versions of each tile were added as controls. All tiles were ordered in one oligo pool from Agilent Technologies. The full list of sequences is provided in Supplementary Tables 1 and 2.

### Motif enrichment and GO analyses

The sequences of all neurite-enriched tiles in N-zip (log_2_FC neurites/soma ≥ 1, adjusted *P* < 0.1; Supplementary Table 1) were used as input for motif discovery and enrichment analysis using the XSTREME web interface^38^. Analysis for enriched functional terms was performed for all genes with a neurite-enriched peak in the N-zip library with gProfiler2 using the default settings^98^. Five top GO terms with the lowest *P* values for biological processes (BPs) and cellular compartments (CCs) domains were used for plotting.

### Quantification and statistical data analysis

Details of exact statistical analyses, packages, tests and other procedures used can be found in the main text, figure legends and Methods. No statistical methods were used to pre-determine sample sizes, but our sample sizes are similar to those reported in previous publications^24–26,53^. Data collection and analysis were not performed blinded to the conditions of the experiments. No datapoint was excluded from the analyses. No randomization was performed. A-parametric tests that do not rely on the assumption of normal distributions were used unless indicated otherwise.

### Reporting summary

Further information on research design is available in the Nature Portfolio Reporting Summary linked to this article.

## Online content

Any methods, additional references, Nature Portfolio reporting summaries, source data, extended data, supplementary information, acknowledgements, peer review information; details of author contributions and competing interests; and statements of data and code availability are available at 10.1038/s41593-022-01243-x.

## Supplementary information

Reporting Summary

Supplementary Table Supplementary Tables 1–7

## Data Availability

Next-generation sequencing data have been deposited at ArrayExpress (accession numbers E-MTAB-10902, E-MTAB-11572 and E-MTAB-11575). The mass spectrometry proteomics data have been deposited to the ProteomeXchange Consortium via the PRIDE^99^ partner repository with the dataset identifiers PXD028300 and PXD026089. smiFISH images have been deposited to the figshare image repository (10.6084/m9.figshare.21196765.v1). Source data are provided with this paper.

## Source data

Source Data Fig. 3 Statistical Source Data
Source Data Fig. 5 Statistical Source Data
Source Data Fig. 5 Unprocessed western blots
Source Data Fig. 7 Statistical Source Data
Source Data Fig. 7 Unprocessed western blots
Source Data Extended Data Fig. 1 Unprocessed western blots
Source Data Extended Data Fig. 2 Statistical Source Data
Source Data Extended Data Fig. 8 Statistical Source Data

## Code availability

The code for N-zip data analysis is available at https://github.com/IgorUlitsky/MPRNA, the code for the SLAM-seq at https://github.com/melonheader/Stability and the code for smiFISH analysis at https://github.com/LauraBreimann/smFISH_neuron_analysis.
