# Supplementary material for: Massively parallel identification of mRNA localization elements in primary cortical neurons
Source: Nat Neurosci. 2023 Jan 16;26(3):394–405. doi: 10.1038/s41593-022-01243-x (PMC9991926; doi:10.1038/s41593-022-01243-x)
Supplement: Supplementary file 1 — Reporting Summary [file 41593_2022_1243_MOESM1_ESM.pdf]

nature portfolio

Corresponding author(s): Marina Chekulaeva, Igor Ulitsky

Last updated by author(s): Nov 16, 2022

Reporting Summary

Nature Portfolio wishes to improve the reproducibility of the work that we publish. This form provides structure for consistency and transparency in reporting. For further information on Nature Portfolio policies, see our Editorial Policies and the Editorial Policy Checklist.

Statistics

For all statistical analyses, confirm that the following items are present in the figure legend, table legend, main text, or Methods section.

n/a	Confirmed	
☐	☒	The exact sample size (*n*) for each experimental group/condition, given as a discrete number and unit of measurement
☐	☒	A statement on whether measurements were taken from distinct samples or whether the same sample was measured repeatedly
☐	☒	The statistical test(s) used AND whether they are one- or two-sided<br>*Only common tests should be described solely by name; describe more complex techniques in the Methods section.*
☒	☐	A description of all covariates tested
☐	☒	A description of any assumptions or corrections, such as tests of normality and adjustment for multiple comparisons
☐	☒	A full description of the statistical parameters including central tendency (e.g. means) or other basic estimates (e.g. regression coefficient) AND variation (e.g. standard deviation) or associated estimates of uncertainty (e.g. confidence intervals)
☐	☒	For null hypothesis testing, the test statistic (e.g. *F*, *t*, *r*) with confidence intervals, effect sizes, degrees of freedom and *P* value noted<br>*Give P values as exact values whenever suitable.*
☐	☒	For Bayesian analysis, information on the choice of priors and Markov chain Monte Carlo settings
☐	☒	For hierarchical and complex designs, identification of the appropriate level for tests and full reporting of outcomes
☐	☒	Estimates of effect sizes (e.g. Cohen's *d*, Pearson's *r*), indicating how they were calculated

Our web collection on statistics for biologists contains articles on many of the points above.

Software and code

Policy information about availability of computer code

Data collection	ENSEMBL biomaRt: https://www.ensembl.org › biomart
Data analysis	N-zip data analysis: https://github.com/IgorUlitsky/MPRNA; PiGx RNA-seq pipeline (v0.0.3): http://bioinformatics.mdc-berlin.de/pigx_docs/pigx-rna-seq.html; SLAM-seq at https://github.com/melonheader/Stability; Max Quant software (v1.6.2.4): https://www.maxquant.org; DeSeq2 (v1.26.0): https://bioconductor.org/packages/release/bioc/html/DESeq2.html; Fiji (2.3.0); RS-FISH (0f6ab4a); Ilastik (v1.4.0); R stringr package (v1.4.0); DEP R (v1.6.1)

For manuscripts utilizing custom algorithms or software that are central to the research but not yet described in published literature, software must be made available to editors and reviewers. We strongly encourage code deposition in a community repository (e.g. GitHub). See the Nature Portfolio guidelines for submitting code & software for further information.

Data

Policy information about availability of data

All manuscripts must include a data availability statement. This statement should provide the following information, where applicable:

- Accession codes, unique identifiers, or web links for publicly available datasets
- A description of any restrictions on data availability
- For clinical datasets or third party data, please ensure that the statement adheres to our policy

NGS data have deposited at the ArrayExpress (accession E-MTAB-10902, E-MTAB-11572, E-MTAB-11575). The mass spectrometry proteomics data have been deposited to the ProteomeXchange Consortium via the PRIDE (Perez-Riverol et al., 2019) partner repository with the dataset identifiers PXD028300 and PXD026089. The smiFISH raw images have been deposited to figshare repository (DOI: https://doi.org/10.6084/m9.figshare.21196765.v1).

Field-specific reporting

Please select the one below that is the best fit for your research. If you are not sure, read the appropriate sections before making your selection.

☒ Life sciences ☐ Behavioural & social sciences ☐ Ecological, evolutionary & environmental sciences

For a reference copy of the document with all sections, see nature.com/documents/nr-reporting-summary-flat.pdf

Life sciences study design

All studies must disclose on these points even when the disclosure is negative.

Sample size	Transcriptomic, proteomic and reporter assays studies were performed in biological triplicates. RT-qPCR analyses were done using biological triplicates and technical duplicates for each biological replicate. The sample size was decided based on pilot experiments.
Data exclusions	No data were excluded.
Replication	Data were successfully reproduced a minimum of three times with similar results. The number of independent biological replicates is indicated in figure legends.
Randomization	Neurons were randomly selected for smiFISH quantification. Randomization was not used in other experiments.
Blinding	Data collection and analysis were not performed blind to the conditions of the experiments. All comparative samples were handled and analyzed in parallel, with the same parameters.

Reporting for specific materials, systems and methods

We require information from authors about some types of materials, experimental systems and methods used in many studies. Here, indicate whether each material, system or method listed is relevant to your study. If you are not sure if a list item applies to your research, read the appropriate section before selecting a response.

Materials & experimental systems

n/a	Involved in the study	
☐	☒	Antibodies
☐	☒	Eukaryotic cell lines
☒	☐	Palaeontology and archaeology
☐	☒	Animals and other organisms
☒	☐	Human research participants
☒	☐	Clinical data
☒	☐	Dual use research of concern

Methods

n/a	Involved in the study	
☒	☐	ChIP-seq
☒	☐	Flow cytometry
☒	☐	MRI-based neuroimaging

Antibodies

Antibodies used	rabbit anti-histone H3 1:5000 (ab1791 Abcam), mouse anti-actin 1:4000 (Sigma A2228), mouse anti-Neurofilament SMI312 1:10000 (837904 BioLegend), rabbit anti-Hbs1l 1:500 (H00010724-PW1 Abnova), mouse anti-Ago2/eIF2C2 1:500 (H00027161-M01 Abnova)
Validation	All antibodies were validated by the suppliers, with cited references on the product page and accurately represented expected expression patterns.<br><br>rabbit anti-histone H3 antibody (ab1791 Abcam) has been validated by Abcam for western blotting with mouse cellular extracts and shown to detect a band of approximately 17 kDa (predicted molecular weight: 15 kDa), as stated on the Abcam product page. This antibody was also validated in our previous work (Zappulo et. al., 2017), shown to detect histone H3 in mouse soma samples via western blotting.<br><br>mouse anti-actin antibody (Sigma A2228) is a monoclonal anti-beta-Actin that recognizes an epitope on the N-terminal end of the b-isoform. The antibody labels specifically beta-actin in a wide variety of tissues and species by various immunochemical techniques, including immunoblotting (42 kDa), as stated on the Sigma product page.<br><br>mouse anti-Neurofilament SMI312 antibody (837904 BioLegend) is a purified monoclonal anti-Neurofilament Marker (pan axonal cocktail) shown to react with human, mouse, and rat protein. It is used for multiple applications, including western blotting, as stated on the BioLegend product page. SMI 312 has been selected to provide a specific marker for axons. This antibody was also validated in our previous work (Zappulo et. al., 2017), shown to detect Neurofilament in mouse neurite samples via western blotting.<br><br>rabbit anti-Hbs1l antibody (H00010724-PW1 Abnova) is a mouse purified polyclonal anti-HBS1L shown to interact with mouse, rat, and human protein. It can be used for Immunoprecipitation and western blotting, as stated on the Abnova product page.

mouse anti-Ago2/eIF2C2 antibody (H00027161-M01 Abnova) is a mouse monoclonal antibody raised against a full-length recombinant EIF2C2 (AAH07633.1). It reacts with mouse, rat, and human protein. It can be used for multiple applications, including western blotting, as stated on the Abnova product.

Eukaryotic cell lines

Policy information about cell lines

Cell line source(s)	HeLa-rtTA cell line was obtained from Kai Schoenig, ZI Mannheim and is describe in Weidenfeld et al. (2009). 293T for lentivirus production were obtained from the MDC.
Authentication	The cell lines behaved as expected in the assays used in the study (let-7 reporter assays, lentivirus production). No further authentication was done.
Mycoplasma contamination	All cell lines were tested negative for mycoplasma contamination.
Commonly misidentified lines (See ICLAC register)	none

Animals and other organisms

Policy information about studies involving animals; ARRIVE guidelines recommended for reporting animal research

Laboratory animals	Male and female Mus musculus embryos (E14) or neonatal pups aged postnatal day 0 (P0) of the C57BL/6J strain were used to prepare cortical neuron cultures. Pregnant mice were individually-housed in cages with standard bedding in a temperature and humidity-controlled room (22-24 °C and 40-60 % humidity) with a 12 h light/12 h dark cycle.
Wild animals	No wild animals were used in this study.
Field-collected samples	No field collected samples were used in this study.
Ethics oversight	All experimental procedures and the handling of mice were conducted according to the policies and regulations established by the Max-Delbrueck-Center for Molecular Medicine (MDC), Germany, and the German regulation authority, das Landesamt für Gesundheit und Soziales (LAGeSo), Berlin. Approval was granted by the responsible Animal Welfare Officer at the MDC, Dr. Claudia Gösele.

Note that full information on the approval of the study protocol must also be provided in the manuscript.
