# Supplementary material for: Massively parallel identification of mRNA localization elements in primary cortical neurons
Source: Nat Neurosci. 2023 Jan 16;26(3):394–405. doi: 10.1038/s41593-022-01243-x (PMC9991926; doi:10.1038/s41593-022-01243-x)
Supplement: Source Data Fig. 5 — Unprocessed western blots [file 41593_2022_1243_MOESM5_ESM.pdf]

Fig. 5d western with markers:

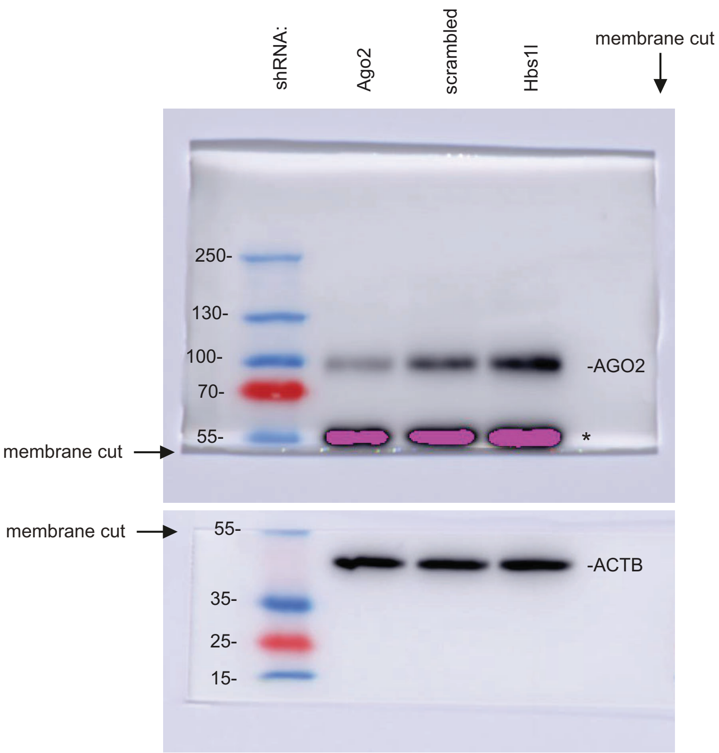

Fig. 5d western without markers:

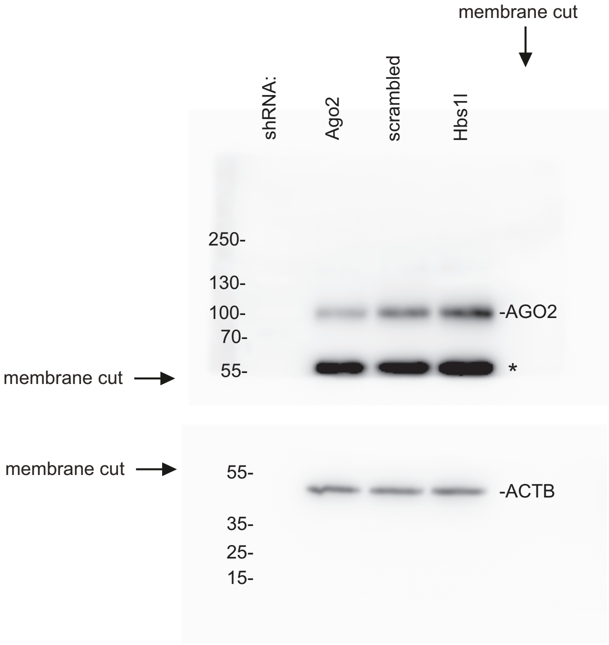

Source Data Fig. 5. Full-size images of western blots shown in Fig. 5d. All samples were run on one gel, membrane was cut into parts, which were incubated with the specified antibodies. * indicates unspecific band detected by anti-AGO2 antibody.
