# Supplementary material for: Massively parallel identification of mRNA localization elements in primary cortical neurons
Source: Nat Neurosci. 2023 Jan 16;26(3):394–405. doi: 10.1038/s41593-022-01243-x (PMC9991926; doi:10.1038/s41593-022-01243-x)
Supplement: Source Data Fig. 7 — Unprocessed western blots [file 41593_2022_1243_MOESM7_ESM.pdf]

Fig. 7d western with markers:

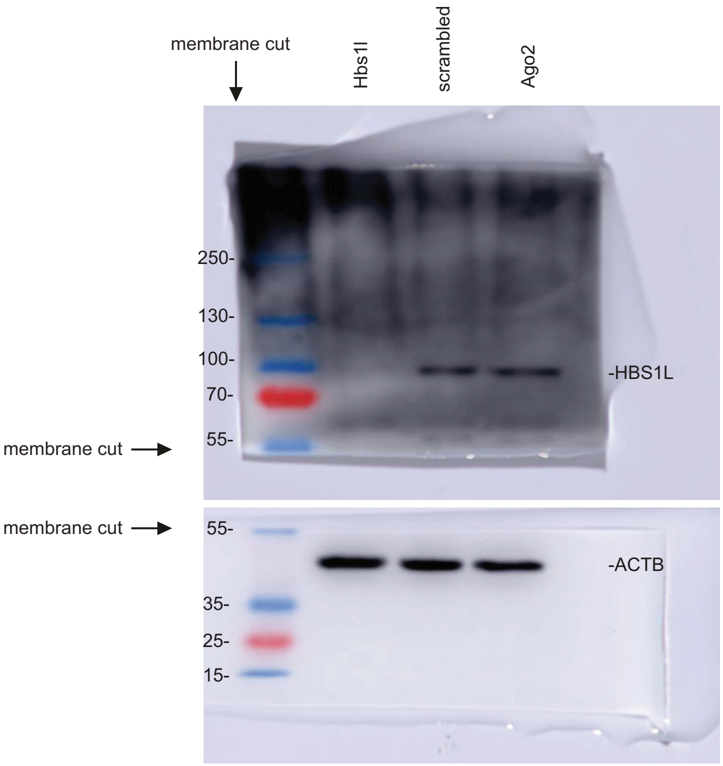

Fig. 7d western without markers:

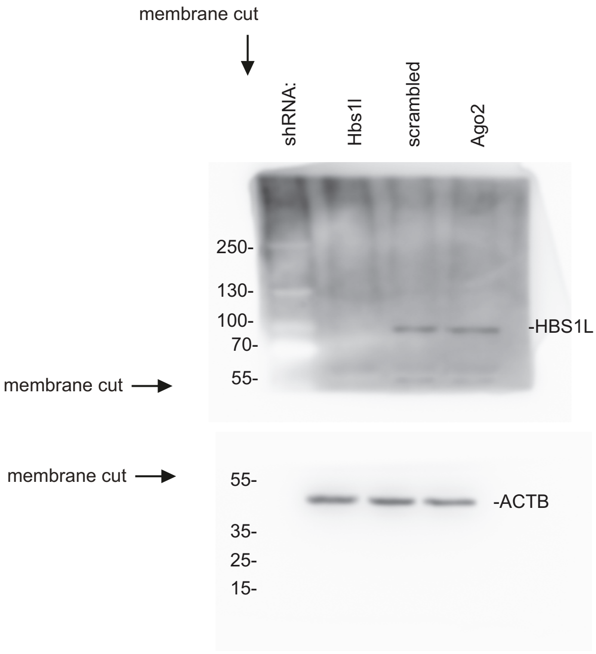

Source Data Fig. 7. Full-size images of western blots shown in Fig. 7d. All samples were run on one gel, membrane was cut into parts, which were incubated with the specified antibodies.
