# Supplementary material for: Massively parallel identification of mRNA localization elements in primary cortical neurons
Source: Nat Neurosci. 2023 Jan 16;26(3):394–405. doi: 10.1038/s41593-022-01243-x (PMC9991926; doi:10.1038/s41593-022-01243-x)
Supplement: Source Data Extended Data Fig. 1 — Unprocessed western blots [file 41593_2022_1243_MOESM8_ESM.pdf]

Extended Data Fig. 1b western with markers:

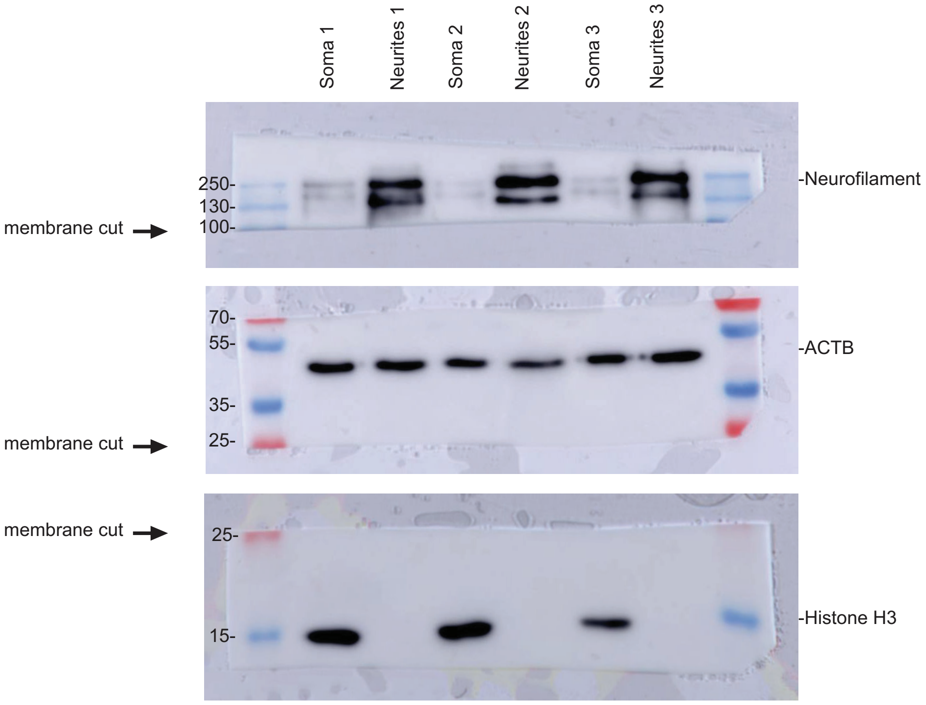

Extended Data Fig. 1b western without markers:

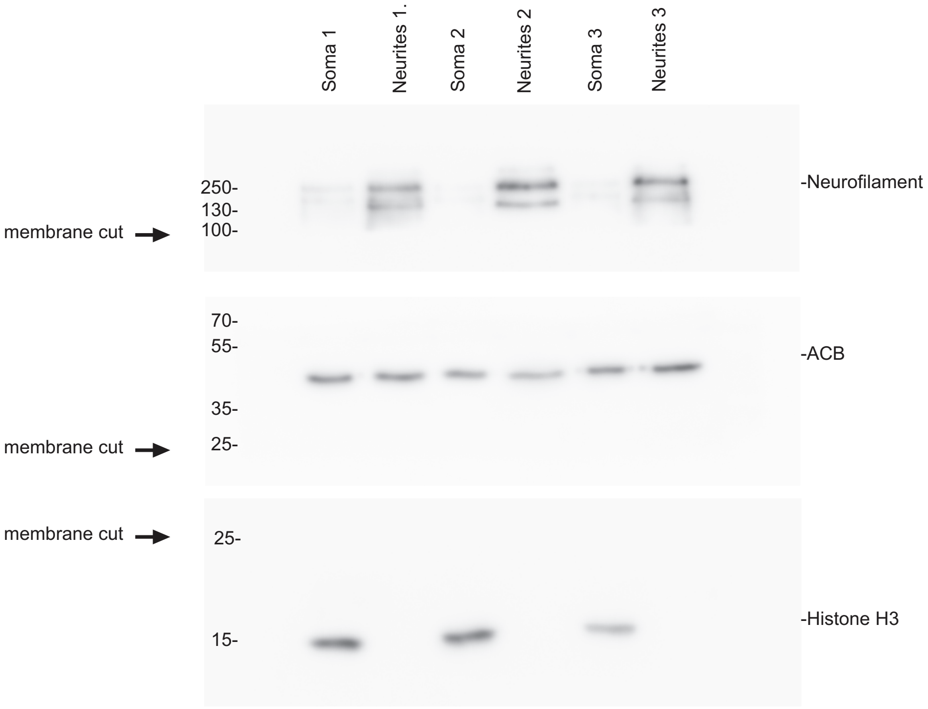

Source Data Extended Data Figure 1. Full-size images of western blots shown in Extended Data Fig. 1b. All samples were run on one gel, membrane was cut into three parts, which were incubated with the specified antibodies.
